# Isolation and Characterization of One New Natural Compound with Other Potential Bioactive Secondary Metabolites from *Glycosmis cyanocarpa* (Blume) Spreng. (Family: Rutaceae)

**DOI:** 10.3390/molecules28052207

**Published:** 2023-02-27

**Authors:** Md. Ariful Islam, Sania Ashrafi, Khondaker Miraz Rahman, Shamim Ahmed, A. H. M. Shofiul Islam Molla Jamal, Monira Ahsan

**Affiliations:** 1 Department of Pharmaceutical Chemistry, University of Dhaka, Dhaka 1000, Bangladesh; 2 School of Cancer and Pharmaceutical Science, King’s College London, 150 Stamford Street, London SE1 9NH, UK; 3 Institute of National Analytical Research and Service (INARS), Bangladesh Council of Scientific and Industrial Research (BCSIR), Dhaka 1205, Bangladesh

**Keywords:** *Glycosmis cyanocarpa*, NMR, penangin, β-Caryophyllene oxide, cytotoxicity, DPPH, phytochemical

## Abstract

*Glycosmis cyanocarpa* (Blume) Spreng is a plant in the Rutaceae family and a species in the Glycosmis genus that has received little attention. Therefore, this research aimed to report the chemical and biological analysis of *Glycosmis cyanocarpa* (Blume) Spreng. The chemical analysis involved the isolation and characterization of secondary metabolites through an extensive chromatographic study, and the structures of these metabolites were elucidated on the basis of a detailed analysis of NMR and HRESIMS spectroscopic data and by comparison with those of related compounds reported in the literature. Different partitions of the crude ethyl acetate (EtOAc) extract were evaluated for antioxidant, cytotoxic, and thrombolytic potentials. In chemical analysis, one new phenyl acetate derivative, namely 3,7,11,15-tetramethylhexadec-2-en-1-yl 2-phenylacetate (1), along with four known compounds N-methyl-3-(methylthio)-N-(2-phenylacetyl) acrylamide (2), penangin (3), β-Caryophyllene oxide (4), and acyclic diterpene-phytol (5) were isolated for the first time from the stem and leaf of the plant. The ethyl acetate fraction showed significant free radical scavenging activity with an IC_50_ value of 11.536 µg/mL compared to standard ascorbic acid (4.816 µg/mL). In the thrombolytic assay, the dichloromethane fraction showed the maximum thrombolytic activity of 16.42% but was still insignificant compared to the standard streptokinase (65.98%). Finally, in a brine shrimp lethality bioassay, the LC_50_ values of dichloromethane, ethyl acetate, and aqueous fractions were found to be 0.687 µg/mL, 0.805 µg/mL, and 0.982 µg/mL which are significant compared to the standard vincristine sulfate of 0.272 µg/mL.

## 1. Introduction

Nature has been the ultimate harbor for humans from the very beginning of civilization. From foodstuffs to therapeutics and livelihood to luxury, civilization has invariably relied on nature. In recent years, the consumption of natural products as dietary supplements for disease prevention or as alternative/complementary medicines (CAM) for disease treatment has become increasingly popular. The biological and molecular diversity of natural products makes it possible to serve as novel templates for future drug design and structural alterations, resulting in more effective and safer medications [1]. Along with technological advancement, natural products are being screened and analyzed more efficiently than ever. Thus, natural products are likely to continue to be the best sources of new commercially viable drug leads.

The family Rutaceae and particularly the genus *Glycosmis* under the family is an exclusive repository of numerous secondary metabolites which are pharmacologically and biologically active, such as flavonoids, alkaloids, phenolic glycosides, quinones, furoquinolines, terpenoids, sulfur-containing amides, gums, reducing sugars, tannins, saponins, acridone, carbazole, and glycerides isolated from diverse plants of the Rutaceae family [2,3,4,5,6]. *Glycosmis cyanocarpa* (Blume) Spreng. is a member of the Rutaceae family. This is a little shrub or tree. Flowers are generally 4-merous, have eight stamens, and have four ovules per locule. Fruits are longer than wide, rectangular, ellipsoid, or ovoid. The plant is found in India, Nepal, South Tibet, Bangladesh, Burma, Sri Lanka, Thailand, Malaysia, the Philippines, and Indonesia (western) [7]. Quinolone alkaloids, cinnamides, nor-diterpenes, and sulfur-containing amides with antifungal activities were isolated from *Glycosmis cyanocarpa* [8,9,10,11,12]. 

Reactive oxygen species (ROS) are formed when there is an imbalance between the defense provided by the antioxidant system and the production of ROS, which leads to the oxidation of lipids, blood vessel walls, carbohydrates, DNA, and other substances [13]. The second-worst cause of death in the world, after cardiovascular disorders, is cancer. Around the world, 182 out of every 100,000 people battle cancer annually, and 102 people lose their lives to the disease. According to a World Health Organization (WHO) report, there are 14 million people globally who are living with cancer, and there are 8 million fatal cases [14]. Blood clots (thrombus) formed in the arteries caused by homeostasis lead to coronary blockage and severe consequences, including acute myocardial and brain infarction, in thrombolytic conditions, which may lead to death. In a procedure called thrombolysis, thrombolytic drugs are used to remove blood clots. The widely used thrombolytic agents for clot breakdown include anistreplase, alteplase, tissue plasminogen activator (tPA) streptokinase, and urokinase [15]. For the situations mentioned above, there are numerous therapy options. However, the use of conventional therapeutic approaches is restricted by side effects, contraindications, the non-selectivity of chemotherapy medications, toxic reactions, and high costs. As a result, it is anticipated that natural products will be more accessible and have fewer side effects.

In this study, we report the isolation and structure elucidation of one new phenyl acetate derivative along with four known compounds that were isolated from this plant for the first time. In addition, the antioxidant, cytotoxicity, and thrombolytic activities of plant extracts were also evaluated and reported following several in vitro approaches.

## 2. Results

### 2.1. Isolated Phytochemicals from G. cyanocarpa

**Figure 1:**
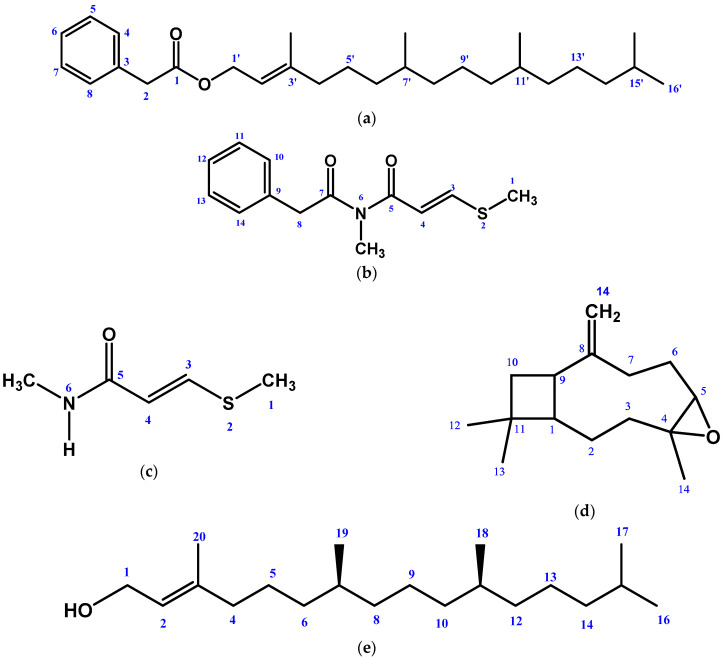
Structures of isolated phytochemicals from *Glycosmis cyanocarpa* using NMR techniques: (**a**) 3,7,11,15-tetramethylhexadec-2-en-1-yl 2-phenylacetate, (**b**) N-methyl-3-(methylthio)-N-(2-phenylacetyl)acrylamide (**c**) Penangin (**d**) β-Caryophyllene oxide (**e**) Phytol.

The EtOAc extract of the stem and leaf parts of *G. cyanocarpa* afforded compounds **1**–**5** (Figure 1) by following repeated chromatographic separations. The structures of the isolated compounds were elucidated as 3,7,11,15-tetramethylhexadec-2-en-1-yl 2-phenylacetate (1), N-methyl-3-(methylthio)-N-(2-phenylacetyl) acrylamide (2), Penangin (3), β-Caryophyllene oxide (4), and phytol (5).

3,7,11,15-tetramethylhexadec-2-en-1-yl 2-phenylacetate (1): Colorless mass and soluble in ethyl acetate and chloroform; ^1^H NMR (400 MHz, CDCl_3_): δ3.62 (2H s, H-2), 7.24–7.30 (m, H-4, 5, 7, 8), 7.3 (m, H-6), 4.61 (2H d, J = 7.2 Hz, H-1′), 5.32 (1H t, J = 7.2 Hz, H-2′), 1.99 (2H t, J = 7.4 Hz, H-4′), 1.24, 1.35 (m, H-5′, 9′), 1.19, 1.28 (m, H-6′,8′), 1.36 (m, H-7′), 1.00, 1.08 (m, H-10′, 12′), 1.36 (m, H-10′, 11′), 1.07, 1.14 (m, H-13′), 1.10, 1.16 (m, H-14′), 1.52 (1H sep, J = 6.8 Hz, H-15′), 0.86 (3H d, J = 6.8 Hz, H-16′), 1.66 (3H s, CH_3_-3′), 0.84 (3H d, J = 6.8 Hz, CH_3_-7′), 0.84 (3H d, J = 6.8 Hz, CH_3_-11), 0.86 (3H d, J = 6.8 Hz, CH_3_-15). ^13^C NMR (100 MHz, CDCl_3_): δ171.7 (C-1), 41.4 (C-2), 134.2 (C-3), 129.3 (C-4,8), 128.5 (C-5,7), 127.0 (C-6), 61.9 (C-1’), 117.9 (C-2’), 143.0 (C-3’), 39.9 (C-4’), 25.1 (C-5’), 37.5 (C-6’), 32.8 (C-7’), 37.4 (C-8’), 24.8 (C-9’), 37.3 (C-10’), 32.7 (C-11’), 37.3 (C-12’), 24.5 (C-13’), 39.4 (C-14’), 28.0 (C-15’), 22.7* (C-16’), 16.4 (CH_3_-3’), 19.8 (CH_3_-7’), 19.7 (CH_3_-11), 22.6* (CH_3_-15).

N-methyl-3-(methylthio)-N-(2-phenylacetyl) acrylamide (2): Colorless mass and soluble in ethyl acetate and chloroform; ^1^H NMR (400 MHz, CDCl_3_): δ 7.90 (1H d, J = 14.4 Hz, H-3), 6.42 (1H d, J = 14.4 Hz, H-4), 4.06 (2H s, H-8), 7.24–7.33 (m, H-10, 12, 14), 7.33 (m, H-11), 7.33 (m, H-13), 3.26 (3H s, N-Me), 2.36 (3H s, S-Me). ^13^C NMR (100 MHz, CDCl_3_): δ149.6 (C-3), 115.1 (C-4), 167.3 (C-5), 174.2 (C-7), 44.3 (C-8), 134.4 (C-9), 129.4 (C-10), 128.6 (C-11), 127.1 (C-12), 128.6 (C-13), 129.4 (C-14), 32.1 (N-Me), 14.9 (S-Me), 167.3 (5-CO), 174.2 (7-CO).

Penangin ((E)-N-methyl-3-methylsulfanylprop-2-enamide) (3): Colorless mass and soluble in ethyl acetate and chloroform; ^1^H NMR (400 MHz, CDCl_3_): δ 2.33 (3H s, H-1) 7.61 (2H d, J = 14.4 Hz, H-3) 5.6 (2H d, J = 14.4 Hz, H-4), 5.4 (1H br q, H-6), (2.89 3H d, N-Me). ^13^C NMR (100 MHz, CDCl_3_): δ14.7 (C-1), 142.9 (C-3), 115.6 (C-4), 165.2 (C-5), 26.4 (N-Me).

β-Caryophyllene oxide (4): Colorless mass and soluble in ethyl acetate and chloroform; ^1^H NMR (400 MHz, CDCl_3_): δ 1.76 (1H m, H-1), 1.42, 1.64 (2H m, H-2), 0.96, 2.08 (2H m, H-3), 2.87 (1H dd, J = 10.5, 4.1, H-5), 1.32, 2.24 (2H m, H-6), 2.12, 2.34 (2H m, H-7), 2.60 (1H q, J = 9.4, H-9), 1.63, 1.69 (2H m, H-10), 1.00 (3H s, H-12) 0.98 (3H s, H-13), 1.20 (3H s, H-14), 4.96, 4.84 (2H s H-15). ^13^C NMR (100 MHz, CDCl_3_): δ50.8 (C-1), 27.0 (C-2), 39.2 (C-3), 59.6 (C-4), 63.8 (C-5), 31.9 (C-6), 30.2 (C-7), 151.7 (C-8), 48.8 (C-9), 39.8 (C-10), 33.9 (C-11), 21.7 (C-12), 29.7 (C-13), 17.0 (C-14), 112.7 (C-15).

Phytol ((2E,7R,11R)-3,7,11,15-tetramethyl-2-hexadecen-1-ol) (5): Colorless mass and soluble in ethyl acetate and chloroform; ^1^H NMR (400 MHz, CDCl_3_): δ4.15 (2H d, J = 6.8 Hz, H-1), 5.42 (1H t, J = 6.8 Hz, H-2), 1.98 (2H t, J = 7.0 Hz, H-4), 0.86 (3H d, J = 6.4 Hz, H-16), 0.86 (3H d, J = 6.4 Hz, H-17), 0.85 (3H d, J = 6.0 Hz, H-18), 0.84 (3H d, J = 6.8 Hz, H-19), 1.66 (3H s, H-20). 

The ^1^H NMR spectrum (400 MHz, CDCl_3_) of Compound **1** displayed five aromatic proton multiplets at δ 7.24–7.30, which can be assigned as H-4, H-5, H-6, H-7, and H-8 of an aromatic ring. The protons directly adjacent to the aromatic ring, i.e., the benzylic proton, resonated at δ 3.62 as a singlet. A de-shielded two proton doublet, indicating its attachment to an oxygen atom, and an olefinic proton triplet appeared at δ 4.61 and δ 5.32. The spectrum further showed a de-shielded methyl signal at δ 1.66 (3H s) and four aliphatic methyl doublets at δ 0.84 (6H d) and 0.86 (6H d) with a coupling constant of 6.8 Hz each.

The ^13^C NMR spectrum showed 28 carbons altogether, including an ester carbonyl group at δ 171.7, to which the benzylic protons at δ 3.62 showed a ^2^J correlation, thus placing the position of the carbonyl carbon at C-1 and benzylic proton at C-2. The benzylic proton further showed a ^3^J correlation to 134.2 (C-3) and 129.3 (C-4, 8). The de-shielded methyl signal at δ 1.66 directly correlated (^1^J) to the carbon at δ 16.4 in the HSQC experiment. 

**Figure 2:**
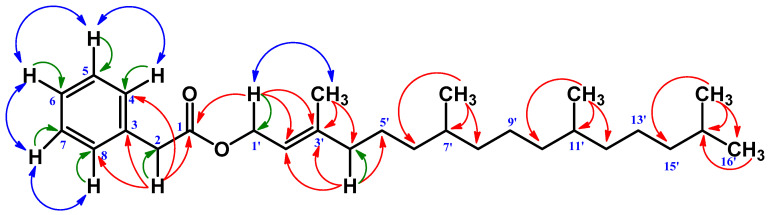
Key HMBC (Red), HSQC (Green), and COSY (Blue) correlation of Compound **1**.

The HMBC spectrum showed a ^2^J correlation to the carbon at δ 143.0 (C-3′) and a ^3^J correlation to δ 117.9 (C-2′) and δ 39.9 (C-4′), confirming its position as CH3-3′. The two methyl doublets at δ 0.84 (6H) showed HMBC correlation to δ 32.8 (C-7′), δ 37.4 (C-8′), 37.3 (C-10′), and 32.7 (C-11′), respectively and thus could be assigned as CH3-7′ and CH3-11′. The rest of the two methyl doublets at δ 0.86 (6H) showed HMBC correlation to δ 39.4 (C-14′) and δ 28.0 (C-15′) only to confirm their position as the terminal two methyls at 15′. The COSY spectrum showed all the expected coupling correlations (Figure 2) (Table 1). The molecular formula of Compound **1** was determined by HRESIMS as C_28_H_46_O_2_, measured in the positive ion mode (*m*/*z* 415.3564 [M + H]^+^). On the basis of this, the above analysis of Compound **1** was identified as 3,7,11,15-tetramethylhexadec-2-en-1-yl 2-phenylacetate. This is a new natural compound reported for the first time from *Glycosmis cyanocarpa*. (Supplementary Materials: Figure S1–S7).

The ^1^H NMR spectrum (400 MHz, CDCl_3_) of Compound **2** showed five aromatic protons at δ 7.24–7.33 as multiplet, which could be assigned as H-10, H-11, H-12, H-13, and H-14 in the aromatic ring. The proton was directly adjacent to the aromatic ring and resonated at δ 4.06 (H-8) as a singlet. The spectrum also showed two olefinic protons at δ 7.90 and δ 6.42 with a coupling constant of J = 14.4 Hz. They can be assigned as H-3 and H-4 protons, respectively. In addition, the spectrum showed N-methyl and S-methyl at δ 3.26 (3H s) and δ 2.36 (3H s), respectively. The ^13^C NMR spectrum (100 MHz, CDCl3) indicated fifteen carbons, including an S-methyl carbon at δ 14.9 and an N-methyl carbon at δ 32.1. 

**Figure 3:**
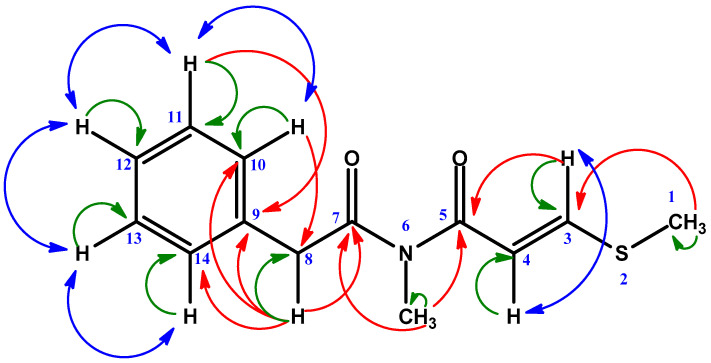
Key HMBC (Red), HSQC (Green), and COSY (Blue) correlation of Compound **2**.

S-methyl showed an HMBC correlation to C-3 (δ 149.6) and N-methyl showed C-5 (δ 167.3), and C-7 (δ 174.2): both C-5 and C-7 were carbonyl carbon. The carbon at δ 149.6 and δ 115.1 showed HMBC correlation to S-methyl (δ 14.9), C-5 (δ 167.3), and C-3 (δ 149.6) protons, respectively. Thus, their positions were confirmed as C-3 and C-4, respectively. Further, the carbon at δ 44.3 showed HMBC correlation to C-10, 14 (δ 128.6), and C-7 (δ 174.2) protons only to confirm its position as C-8. The HSQC and COSY spectra showed all the expected coupling correlations (Figure 3) (Table 2). On this basis, Compound **2** was identified as N-methyl-3-(methylthio)-N-(2-phenylacetyl) acrylamide. (Figure S8–S12).

In the ^1^H NMR spectrum (400 MHz, CDCl_3_) of Compound **3**, the signal at δ 2.89 was indicative of an N-Me group with a coupling to N-H (H-6) where the N-H signal was evident at δ 5.4 as a broad quartet. The sharp singlet at δ 2.33 must have been due to an X-Me group (X can be either CO, O, or S); here, the chemical shift was in favor of S-Me according to the previously published data in [11]. The chemical shifts at δ 7.60 and δ 5.60 with a coupling constant of J = 14.4 Hz were characteristic of the olefinic proton and assigned as H-4 and H-3 in the structure.

The ^13^C NMR spectrum (100 MHz, CDCl_3_) was also in agreement with the structure. Only five signals were observed. One Me at δ 14.7 was typical for S-Me (C-1). Another Me at δ 26.4 was assigned N-Me. Two olefinic carbon atoms, C-4 and C-3, were observed at δ 115.6 and δ 142.9. Finally, the C=O carbon was found to resonate at δ 165.2. All the data were found in close correlation with those reported in the previous publication (Table 3) [11]. (Figures S13 and S14).

The ^1^H NMR spectrum (400 MHz, CDCl_3_) of Compound **4** displayed three characteristic methyl protons at δ 1.00, 0.98, and 1.20 as a singlet with the integration value of three, which were assignable to H-12, H-13, and H-14, respectively. The exocyclic methylene proton appeared at δ 4.96 and 4.84 and was assigned to H-15. The proton present in the epoxide ring (H-5) was assured as it resonated at δ 2.87 as a double doublet. The proton signal at δ 1.76, 2.60, and 1.63–1.69 appeared as a multiplet and was assignable to H-1, H-9, and H-10, respectively, in the cyclobutane ring. The remaining signals of the spectrum showed similar resonance as those described in [16]. 

The ^13^C NMR spectrum (100 MHz, CDCl_3_) indicated fifteen carbons, including a methylene group (δ 112.7, C-15) attached to an exocyclic double bond, and three methyl groups: at δ 21.7 (C-12), δ 29.7 (C-13), and δ 17 (C-14). The carbon in the cyclobutene ring appeared at δ 50.8, 48.8, 39.8, and 33.9, only to be assigned as C-1, C-9, C-10, and C-11, respectively. δ 59.6 and δ 63.8 represented two carbons in the epoxide ring at C-4 and C-5. The rest of the signals appeared at δ 27, 39.2, 31.9, 30.2, and 151.7, only to be assigned as C-2, C-3, C-6, C-7, and C-8 as described in the previously published data [16] (Table 4). Thus, Compound **4** was confirmed to be β-Caryophyllene oxide. (Figure S15).

The ^1^H NMR spectrum (400 MHz, CDCl_3_) of Compound **5** displayed an oxygenated methylene doublet at δ 4.15 (2H d, J = 6.8 Hz), an olefinic proton at δ 5.42 (1H t, J = 6.8 Hz), and a deshielded methyl at δ 1.66 (3H s) which could be assigned to H-1, H-2, and H-20 of an acyclic diterpene, Phytol. In addition, the spectrum showed four methyl doublets (3H d) at δ 0.86 (J = 6.4 Hz), 0.86 (J = 6.4 Hz), 0.85 (J = 6.0 Hz), and 0.84 (J = 6.8 Hz) which were assignable to H-16, H-17, H-18, and H-19. A broad triplet at δ 1.98 (2H t, J = 7.0 Hz) was assigned as H-4. The 1H NMR data were found to be in close agreement with those published for the compound [17,18]. All these data permitted the identification of Compound **5** as Phytol (Table 5) (Figure S16).

### 2.2. Effect of G. cyanocarpa Extracts on DPPH Free Radical Scavenging Activity

**Figure 4:**
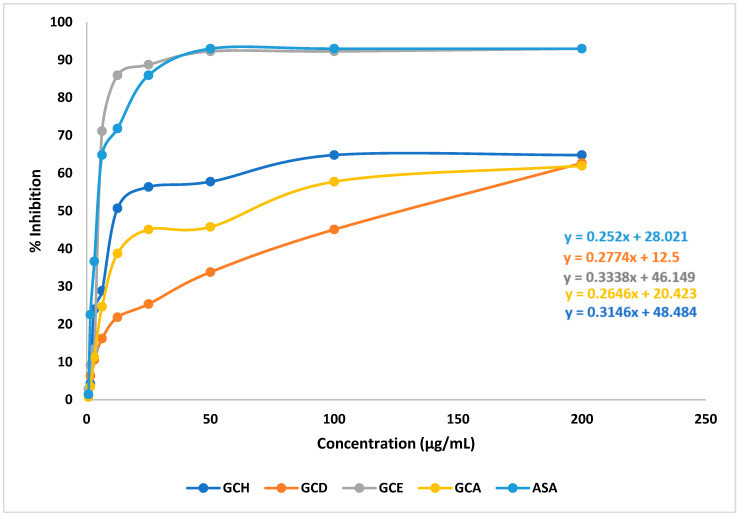
Linear regression equations (IC_50_) of ascorbic acid (ASA) and different extracts of *Glycosmis cyanocarpa*.

In the DPPH free radical scavenging study, different extracts of *G. cyanocarpa* exhibited a dose-dependent free radical scavenging activity in comparison with the standard. GCE showed substantial scavenging activity (92.96%) compared to the standard ASA (92.96%) at 200 µg/mL. The IC_50_ values of ASA and the fractions were calculated by the linear regression equation, as summarized in Figure 4.

### 2.3. Effect of G. cyanocarpa Extracts on Brine Shrimp Lethality Bioassay

**Figure 5:**
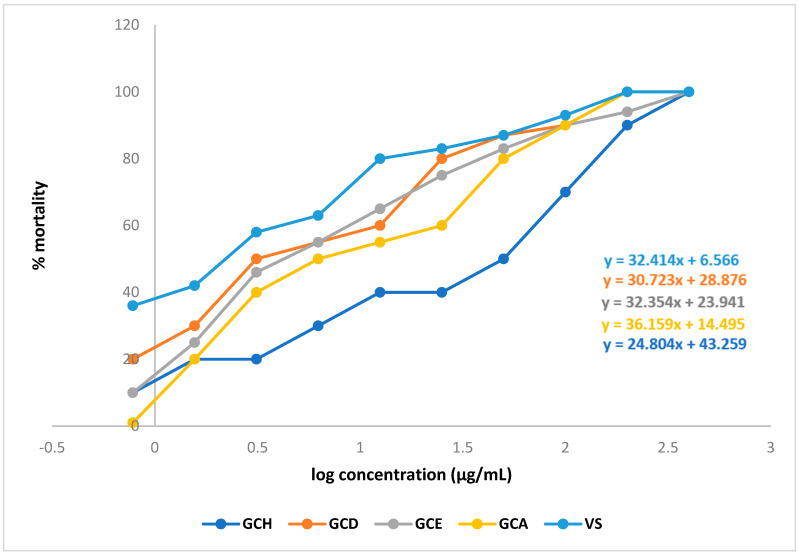
Linear regression equations (LC_50_) of vincristine sulphate (VS) and different extracts of *Glycosmis cyanocarpa*.

LC_50_ values of the different fractions were 0.687 µg/mL (GCD), 0.805 µg/mL (GCE), and 0.982 µg/mL (GCA). By comparing the LC_50_ values with the standard (0.272 µg/mL), it can be said that all the fractions showed significant cytotoxic activity. (Figure 5) (Table 6).

### 2.4. Effect of G. cyanocarpa Extracts on the Thrombolytic Activity

The DCM fraction of the ethyl acetate extract (GCD) of *Glycosmis cyanocarpa* showed maximum thrombolytic activity around 16.42%, but it was not significant compared to the standard (65.98%). However, the other fractions showed a much lower percentage of lysis activity (Table 7).

## 3. Discussion

Since the dawn of human civilization, various diseases have been treated with medications and chemicals derived from plants. Since the dawn of time, all civilizations have made considerable use of plants to enhance health and treat a variety of ailments. According to the WHO, traditional medicine is the primary source of health care for 80% of people worldwide [19]. 

Phenylacetate, a common metabolite of phenylalanine, is found naturally in human plasma and acts as an endogenous growth regulator in plants [20]. In the previous literature, phenylacetate derivatives were reported to show substantial biological activities such as cytotoxic, antifungal, hypnotic, and wound-healing properties [21,22,23,24,25,26]. Moreover, prenylated compounds with one or more prenyl moieties are common natural products that have been isolated predominantly from nature. These compounds have attracted much attention because they often possess antimicrobial, antioxidant, anti-inflammatory, antiviral, and anticancer activities [27,28]. These activities depend on the length of the side chain and the nature and relative position of substituent groups on the aromatic ring [16]. The discovery of 3,7,11,15-tetramethylhexadec-2-en-1-yl 2-phenylacetate (1) is an important addition to the diverse array in the rapidly expanding class of phenylacetate in terms of both chemical structure and biological activities. 

Sulphur-containing amides and cinnamides with antifungal activities have been isolated from *Glycosmis cyanocarpa* in previous studies [10,12]. From the structural similarities between previously isolated amides and N-methyl-3-(methylthio)-N-(2-phenylacetyl) acrylamide (2), a concrete assumption can be portrayed that Compound **2** can possess a prospective antifungal activity. Penangin (3) has reported antifungal activity, and the compound has been previously isolated from different species of *Glycosmis chlorosperma* [11]. Terpenoids, also known as isoprenoids, are the most numerous and structurally diverse natural products found in many plants. Several studies, including in vitro, preclinical, and clinical, have confirmed that this class of compounds displays a wide array of very important pharmacological properties [29]. Sesquiterpene β-Caryophyllene oxide (4) was isolated previously from other plant sources. This compound is popular for its significant anticancer, antifungal, insecticidal, analgesic, and inflammatory activities [30,31,32,33,34]. Acyclic diterpene phytol (5) has been depicted in numerous studies as a potent antibacterial, cytotoxic, antioxidant, anticonvulsant, antinociceptive, anti-inflammatory, and immune-modulating agent [35,36,37,38,39]. The biological activities of different fractions of crude ethyl acetate extracts of *Glycosmis cyanocarpa* have also demonstrated the potential of the plant to be an important asset in terms of natural medicine.

As mentioned above, *Glycosmis cyanocarpa* proved to be a rich source of biologically active secondary metabolites. Extensive investigations on the isolated compounds are still urged to determine their exact mode of action and to exert a pharmacological response along with their safety profile. Large-scale studies are also required to isolate other bioactive phytochemicals from this potentially medicinal plant.

## 4. Materials and Methods

### 4.1. Collection and Preparation of the Plant Material

The stem and leaf parts of *Glycosmis cyanocarpa* were collected from Kaptai, Rangamati, Bangladesh, in March 2021. A voucher specimen was deposited in the National Herbarium, Dhaka, Bangladesh, with the accession number DACB 65592. The plant parts were thoroughly cleaned. The portions were cut into little pieces and dried for several weeks in the shade. The dried material was then carefully crushed into a coarse powder using a high-capacity grinding machine. The final product sample was 1.3 kg.

### 4.2. Instrumentations, Drugs, and Chemicals

The Bruker (400 MHz) instrument was used to record NMR spectra in deuterated chloroform (CDCl_3_). Solvent evaporation was performed by Buchi Rotavapor (Essen, Germany). Kieselgel 60H and Sephadex LH 20 (Sigma-Aldrich, St. Louis, MI, USA) were used to perform vacuum liquid chromatography (VLC) and gel permeation chromatography (GPC), respectively. An analysis of the compounds was performed on precoated thin layer chromatography (PTLC) plates (Silica gel 60 F 254, Darmstadt, Merck, Germany). UV light and vanillin/H_2_SO_4_ reagents were used for the visualization of the spots on TLC (thin layer chromatography) plates. All the other reagents and solvents consumed in the research were of an analytical grade and obtained from a reliable source (Active Fine Chemicals Ltd., Bangladesh; Merck, Germany; DaeJung, Republic of Korea). Streptokinase, ascorbic acid, and vincristine sulfate used in the study were obtained from Opsonin Pharma Ltd., Dhaka, Bangladesh.

### 4.3. Experimental Design

#### 4.3.1. Extraction of Plant Material

About 1.3 kg of *Glycosmis cyanocarpa* (500 g stem and 790 g leaf) was taken in clean, amber-colored bottles and soaked in distilled EtOAc for about 3–4 weeks with occasional shaking and stirring. After cold extraction, the whole mixture was filtered by a cotton plug in a large funnel, and the volume of the filtrate was then reduced using a Buchii Rotavapour. This process was carried out multiple times, and dried extracts were collected in the same beaker. Through extraction, the ethyl acetate soluble fraction of the crude extract was separated. The weight of the cumulative extract of *Glycosmis cyanocarpa* was 48 g (3.69%).

#### 4.3.2. Isolation of Compounds

The extract was fractionated by vacuum liquid chromatography using petroleum ether, EtOAc, and MeOH with increasing polarity [40]. A total of 43 fractions were collected. VLC fractions 10 and 11 (5% hexane in toluene) were fractionated on a Sephadex LH-20 column into 20 fractions, each using CHCl_3_ as the eluting solvent. Sephadex fractions 9–16 showed a purple spot with vanillin/H_2_SO_4_ after heating. These fractions were subjected to preparative TLC (silica gel; 5% hexane in toluene, multiple developments) to obtain Compound **1** (7.8 mg, Rf 0.89, 5% hexane in toluene). VLC fractions 5 (30% toluene in hexane)) and 29 (50% ethyl acetate in hexane) were conducted on a Sephadex LH-20 column, and compounds **2**, **3**, **4**, and **5** were isolated from sephadex fractions 13–16, 13–17, 7–15, and 13–18 of VLC fractions 10–11, 5, 29, and 2, respectively.

#### 4.3.3. Preparation of Different Partitions for Biological Tests

The solvent-solvent partitioning was carried out according to a methodology developed by Kupchan and modified by Van Wagenen et al. [41]. With n-hexane, dichloromethane (DCM), and ethyl acetate, the crude EtOAc extract was fractionated. The n-hexane soluble fraction (GCH, 1.7 g), DCM soluble fraction (GCD, 1.3 g), ethyl acetate soluble fraction (GCE, 1.2 g), and aqueous soluble fractions (GCA, 0.7 g) were then produced by separately evaporating each of these fractionates in a Rotary evaporator.

#### 4.3.4. Structural Identification of the Compounds

The Bruker 400 NMR spectrometer and HRESIMS were used to measure the ^1^H NMR spectra of compounds **1**–**5** in deuterated chloroform (CDCl_3_) at 400 MHz, and the δ values are described relative to the residual non-deuterated solvent signal. Coupling constants are given in Hertz (Hz). The chemical shifts are expressed in δppm.

### 4.4. Antioxidant Assay

#### DPPH Free Radical Scavenging Assay

A total of 3.0 mL of a DPPH methanol solution (20 μg/mL) and 2.0 mL of a plant extract solution at serially diluted different concentrations (200 μg/mL to 0.78125 μg/mL) were combined to test the ability of plant extracts to scavenge free radicals on 1, 1-diphenyl-2-picrylhydrazyl (DPPH). The decolorizing of a purple DPPH methanol solution by the plant extract was compared to that of ascorbic acid (ASA) to determine antioxidant capabilities [17,42,43,44].

$$\%\;Inhibition\;of\;free\;radical\;DPPH=\left(1-\frac{Absorbance\;of\;sample}{Absorbance\;of\;the\;control\;reaction}\right)\times 100$$

### 4.5. Cytotoxicity Assay

#### Brine Shrimp Lethality Bioassay

To assess the potential cytotoxicity of various plant extracts, the brine shrimp lethality test was carried out. To simulate seawater, 38 g of NaCl salt was dissolved in 1000 mL of distilled water along with NaOH to maintain a constant pH. (8.0). In artificial saltwater, brine shrimp eggs were incubated to develop into nauplii. Before being introduced to the test samples, dimethyl sulfoxide (DMSO) was diluted in several steps to different concentrations (400 μg/mL to 0.78125 μg/mL ). Vincristine sulfate was used as the reference standard in a range of dosages (400 μg/mL to 0.78125 μg/mL ), and DMSO was used as the adverse control. A total of 5 mL of simulated saltwater was added to vials of the mixture after the nauplii were counted visually [17,42,44,45].

$$Mortality\left(\%\right)=\frac{Number\;of\;nauplii\;death}{Number\;of\;nauplii\;taken}\times 100$$

### 4.6. In Vitro Thrombolytic Assay

The approach outlined by Ahmed et al. was used to conduct this investigation [46]. A total of 10 mL of venous blood from healthy volunteers was taken. Blood was supplied in a pre-weighed, sterile Eppendorf tube at a rate of 0.5 mL/tube. They formed clots after 45 min of incubation at 37 °C. All of the produced serum was removed except for the clot. To calculate the clot weight, each tube was once again weighed.

**Clot weight = weight of clot-containing tube − the weight of tube alone**

Separately, 100 μL of the extract solutions were added to the tubes. A total of 100 μL of streptokinase was added separately as a positive control. Separately, 100 μL of distilled water was added to the collection of empty tubes. For 90 min, all of the tubes were incubated at 37 °C. The difference obtained in weight taken before and after clot lysis was expressed as the percentage of clot lysis as shown below:

**% clot lysis = (Weight of the clot after lysis/Weight of clot before lysis) × 100**

## 5. Conclusions

In this study, GCE showed the maximum free radical scavenging activity, while GCD, GCE, and GCA exhibited substantial cytotoxic activity. No fractions of the plant showed any significant thrombolytic activity. The ethyl acetate extract of the whole plants of *G. cyanocarpa,* upon successive chromatographic separation and purification, yielded a total of five compounds, including one new natural compound. It is still necessary to conduct in-depth research on this plant to identify potential bioactive phytochemicals, isolate them, and ascertain their precise pharmacological effects as well as their safety profile.

## Figures and Tables

**Table 1:** NMR spectroscopic data (400 MHz, CDCl_3_) for Compound **1**.

Position	δ_C_	δ_H_	HMBC
1	171.7	---	---
2	41.4	3.62 2H s	171.7 (C-1), 134.2 (C-3), 129.3 (C-4)
3	134.2	---	---
4,8	129.3	7.24–7.30 m	---
5,7	128.5	7.24–7.30 m	---
6	127.0	7.3 m	---
1′	61.9	4.61 2H d (*J* = 7.2 Hz)	171.7 (C-1), 143.0 (C-3′), 117.9 (C-2′)
2′	117.9	5.32 t (*J* = 7.2 Hz)	---
3′	143.0	---	---
4′	39.9	1.99 2H t (*J* = 7.4 Hz)	143.0 (C-3′), 117.9 (C-2′)
5′	25.1	1.35 m, 1.24 m	---
6′	37.5	1.28 m, 1.19 m	---
7′	32.8	1.36 m	---
8′	37.4	1.28 m, 1.19 m	---
9′	24.8	1.35 m, 1.24 m	---
10′	37.3	1.08 m, 1.00 m	---
11′	32.7	1.36 m	---
12′	37.3	1.08 m, 1.00 m	---
13′	24.5	1.14 m, 1.07 m	---
14′	39.4	1.16 m, 1.10 m	22.6* (C-16), 22.7* (15′-CH_3_)
15′	28.0	1.52 1H sep (*J* = 6.8 Hz)	22.6* (C-16), 22.7* (15′-CH_3_)
16′	22.7*	0.86 3H d (*J* = 6.8 Hz)	
CH_3_-3′	16.4	1.66 3Hs	143.0 (C-3′), 117.9 (C-2′), 39.9 (C-4′)
CH_3_-7′	19.8	0.84 3H d (*J* = 6.8 Hz)	32.7 (C-11′), 32.8 (C-7′)
CH_3_-11	19.7	0.84 3H d (*J* = 6.8 Hz)	37.3 (C-10′), 37.4 (C-8′)
CH_3_-15	22.6*	0.86 3H d (*J* = 6.8 Hz)	39.4 (C-14′), 28.0 (C-15′)

* denotes interchangeable chemical shift assignments.

**Table 2:** NMR spectroscopic data (400 MHz, CDCl_3_) for Compound **2**.

Position	δ_C_	δ_H_	HMBC
3	149.6	7.90 d (*J* = 14.4 Hz)	14.9 (S-Me), 167.3 (C-5)
4	115.1	6.42 d (*J* = 14.4 Hz)	149.6 (C-3)
5	167.3	---	---
7	174.2	---	---
8	44.3	4.06 2H s	128.6 (C-10,14)) 174.2(C-7)
9	134.4	---	
10	129.4	7.24–7.33 m	44.3 (C-8)
11	128.6	7.33 m	---
12	127.1	7.24–7.33 m	---
13	128.6	7.33 m	---
14	129.4	7.24–7.33 m	44.3 (C-8)
N-Me	32.1	3.26 3H s	167.3 (C-5), 174.2(C-7)
S-Me	14.9	2.36 3H s	149.6 (C-3)
5-CO	167.3	---	---
7-CO	174.2	---	---

**Table 3:** NMR spectroscopic data (400 MHz, CDCl_3_) for Compound **3**.

Position	Compound-3		Penangin [11]	
	δ_H_	δ_C_	δ_H_	δ_C_
1	2.33 s	14.7	2.32 s	14.6
2	---	---	---	---
3	7.61 d	142.9	7.62 d	142.6
4	5.6 d	115.6	5.61 d	115.7
5	---	165.2	---	165.2
6	5.4 br q	---	5.3 br q	---
N-Me	2.89 d	26.4	2.88	26.3

**Table 4:** NMR spectroscopic data (400 MHz, CDCl_3_) for Compound **4**.

Position		Compound-4	β-Caryophyllene Oxide [16]	
	δ_C_	δ_H_	δ_C_	δ_H_
1	50.8	1.76 m	50.9	1.76 t (*J* = 9.3 Hz)
2	27	1.64 m, 1.42 m	27.1	1.65 m, 1.43 m
3	39.2	0.96 m, 2.08 m	39.2	0.98 m, 2.11 m
4	59.6	---	59.7	---
5	63.8	2.87 dd (*J* = 10.5, 4.1)	63.7	2.87 dd (*J* = 10.6,4.2)
6	31.9	2.24 m, 1.32 m	31.8	2.24 m 1.30 m
7	30.2	2.34 m, 2.12 m	30.2	2.33 ddd *(J* = 4.6, 7.7, 12.4 Hz) 2.11 m
8	151.7	----	151.6	----
9	48.8	2.60 q (*J* = 9.4)	48.7	2.61 q (*J* = 9.3)
10	39.8	1.63–1.69 2H m	39.8	1.65 2H m
11	33.9	---	34.0	---
12	21.7	1.00 3H s	21.6	1.01 3H s
13	29.7	0.98 3H s	29.8	0.98 3H s
14	17.0	1.20 3H s	17.0	1.20 3H s
15	112.7	4.96 s, 4.84 s	112.7	4.98, 4.86 s

**Table 5:** NMR spectroscopic data (400 MHz, CDCl_3_) for Compound **5**.

Position	Compound 5	Phytol [18]
	δ_H_	δ_H_
H-1	4.15, 2H d (*J* = 6.8 Hz)	4.14, 2H d (*J* = 6.8 Hz)
H-2	5.42, 1H t (*J* = 6.8 Hz)	5.40, 1H dq (*J* = 6.8 Hz, 1.4 Hz)
H-4	1.98, 2H t (*J* = 7.0 Hz)	1.99, 2H t (*J* = 7.0 Hz)
H-16	0.86, 3H (*J* = 6.4 Hz)	0.87, 6H d (*J* = 6.3 Hz)
H-17	0.86, 3H d (*J* = 6.4 Hz)	0.87, 6H d (*J* = 6.3 Hz)
H-18	0.85, 3H d (*J* = 6.0 Hz)	0.85, 3H d (*J* = 6.1 Hz)
H-19	0.84, 3H d (*J* = 6.8Hz)	0.84, 3H d (*J* = 6.6 Hz)
H-20	1.66, 3H s	1.66, 3H s

**Table 6:** LC_50_ values of the test sample with *Glycosmis cyanocarpa*.

Test Sample	LC_50_ (µg/mL)
Vincristine Sulphate (VS)	0.272
GCH	1.339
GCD	0.687
GCE	0.805
GCA	0.982

**Table 7:** Thrombolytic activity of different fractions of *Glycosmis cyanocarpa*.

Sample	Weight of Empty Eppendorf Tube (W_1_) g	Weight before Clot Disruption (W_2_) g	Weight after Clot Disruption (W_3_) g	Weight before Clot Lysis (W_4_ = W_2_ − W_1_) g	Weight of Lysis Clot (W_5_ = W_2_ − W_3_) g	% Lysis (W_5_/W_4_) × 100
GCH	0.8176	1.139	1.1207	0.3214	0.0183	5.69
GCD	0.8284	1.0879	1.0453	0.2595	0.0426	16.42
GCE	0.8256	1.2122	1.1993	0.3866	0.0129	3.34
GCA	0.8241	1.152	1.1319	0.3279	0.0201	6.13
Blank	0.8436	1.8425	1.8422	0.9989	0.0003	0.03
SK	0.798	1.427	1.012	0.629	0.415	65.98

## Data Availability

Not Applicable.

## Supplementary Materials

The following supporting information can be downloaded at: 457 https://www.mdpi.com/article/10.3390/molecules28052207/s1, Figure S1: ^1^H NMR spectrum (400 MHz, CDCl_3_) of Compound **1**, Figure S2: Partially expanded ^1^H NMR spectrum (400 MHz, CDCl_3_) of Compound **1**, Figure S3: ^13^C NMR spectrum (100 MHz, CDCl_3_) of Compound **1**, Figure S4: Partially expanded HSQC spectrum (400 MHz, CDCl_3_) of Compound **1**, Figure S5: Partially expanded HMBC spectrum (400 MHz, CDCl_3_) of Compound **1**, Figure S6: Partially expanded COSY spectrum (400 MHz, CDCl_3_) of Compound **1**, Figure S7: HRESIMS Spectrum of Compound **1**, Figure S8: ^1^H NMR spectrum (400 MHz, CDCl_3_) of Compound **2**, Figure S9: ^13^C NMR spectrum (100 MHz, CDCl_3_) of Compound **2**, Figure S10: HSQC spectrum (400 MHz, CDCl_3_) of Compound **2**, Figure S11: HMBC spectrum (400 MHz, CDCl_3_) of Compound **2**, Figure S12: COSY spectrum (400 MHz, CDCl_3_) of Compound **2**, Figure S13: ^1^H NMR spectrum (400 MHz, CDCl_3_) of Compound **3**, Figure S14: ^13^C NMR spectrum (100 MHz, CDCl_3_) of Compound **3**, Figure S15: ^1^H NMR spectrum (400 MHz, CDCl_3_) of Compound **4**, Figure S16: ^1^H NMR spectrum (400 MHz, CDCl_3_) of compound **5**.

## Sample Availability

Samples of the compounds are available from the authors.
