# Supplementary material for: Isolation and Characterization of One New Natural Compound with Other Potential Bioactive Secondary Metabolites from Glycosmis cyanocarpa (Blume) Spreng. (Family: Rutaceae)
Source: Molecules. 2023 Feb 27;28(5):2207. doi: 10.3390/molecules28052207 (PMC10005784; doi:10.3390/molecules28052207)
Supplement: Supplementary file 1 [file molecules-28-02207-s001.zip › molecules-2195105-supplementary.pptx]

## Slide 1
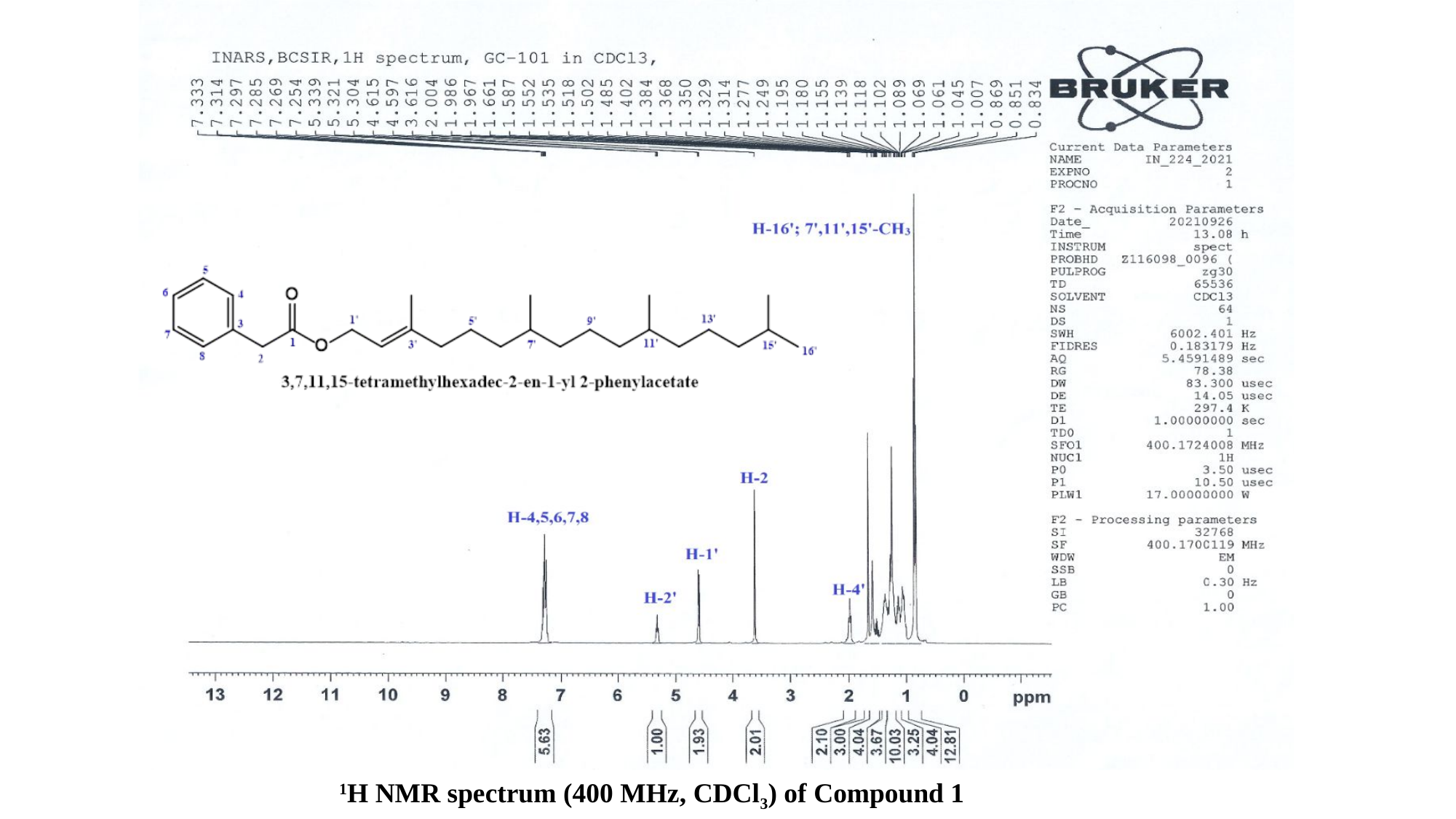

1H NMR spectrum (400 MHz, CDCl3) of Compound 1

## Slide 2
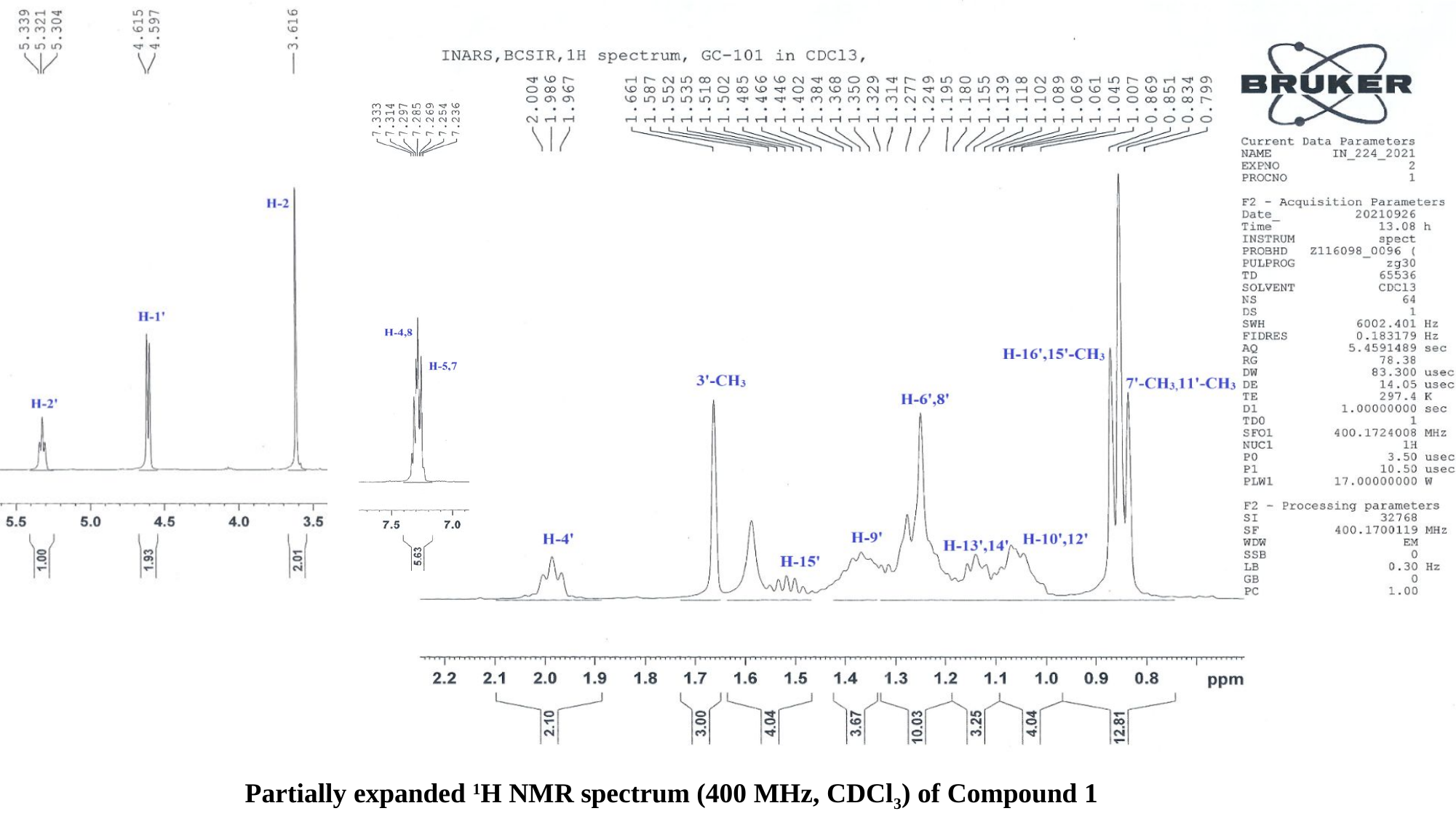

Partially expanded 1H NMR spectrum (400 MHz, CDCl3) of Compound 1

## Slide 3
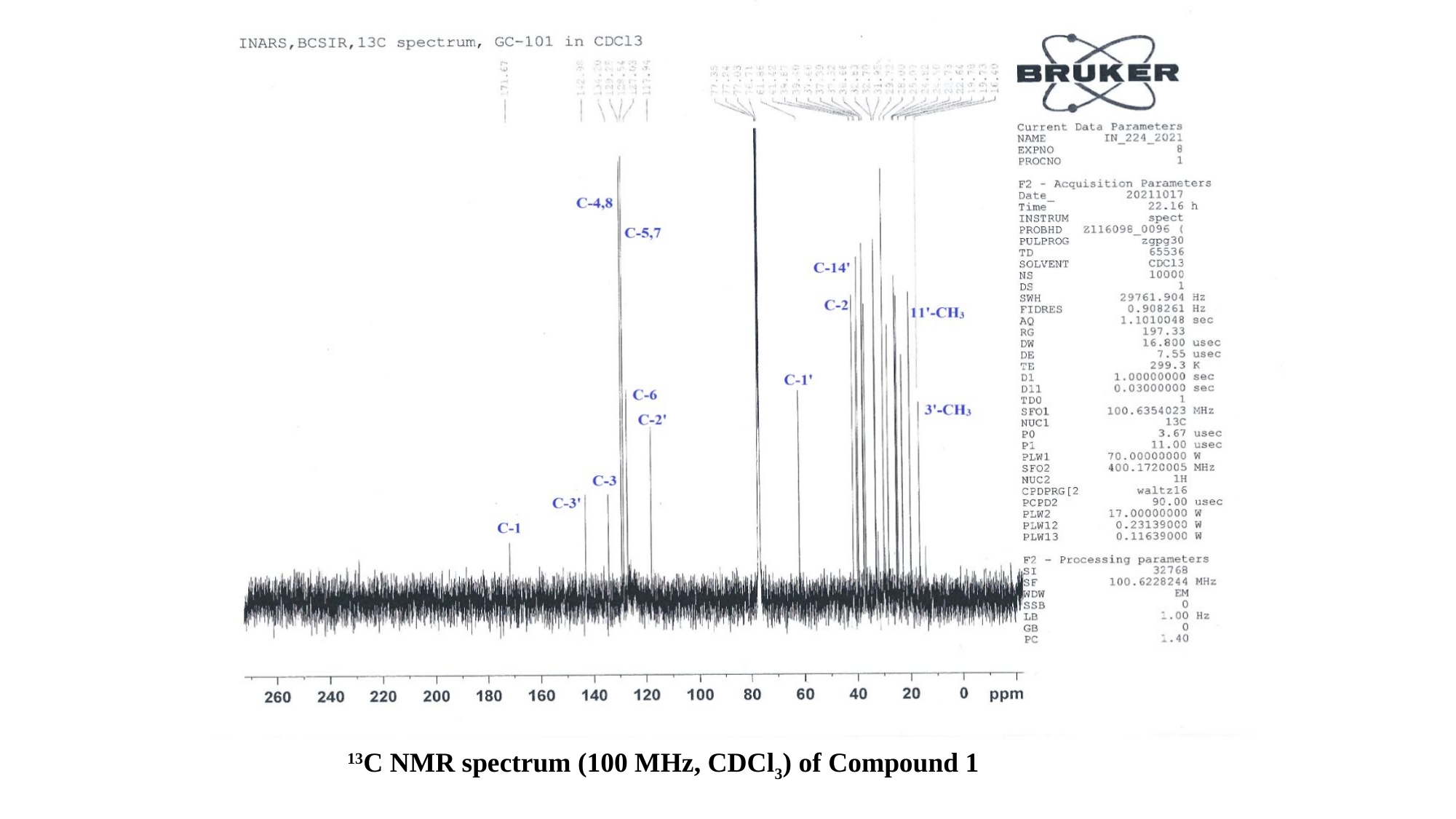

13C NMR spectrum (100 MHz, CDCl3) of Compound 1

## Slide 4
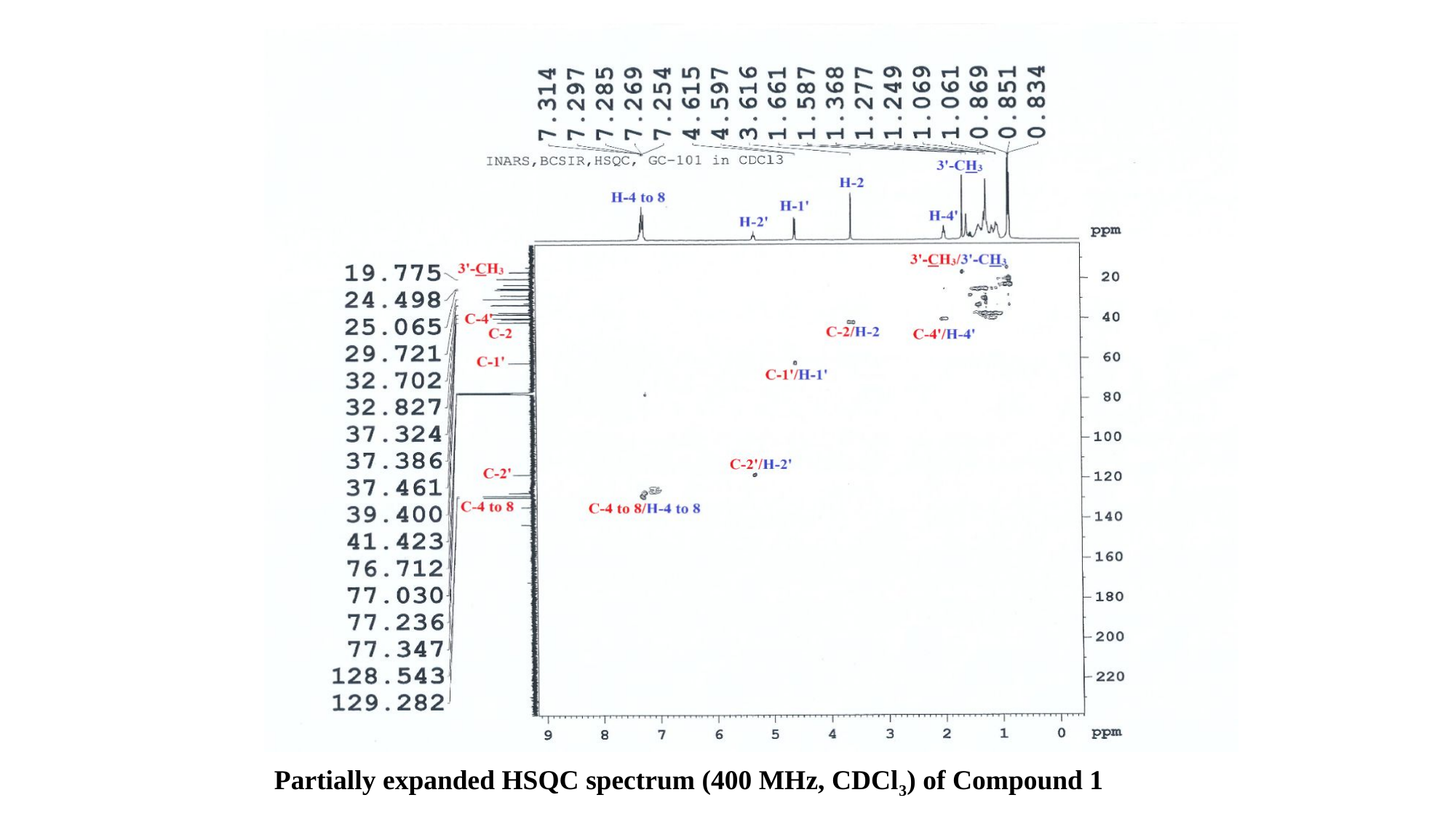

Partially expanded HSQC spectrum (400 MHz, CDCl3) of Compound 1

## Slide 5
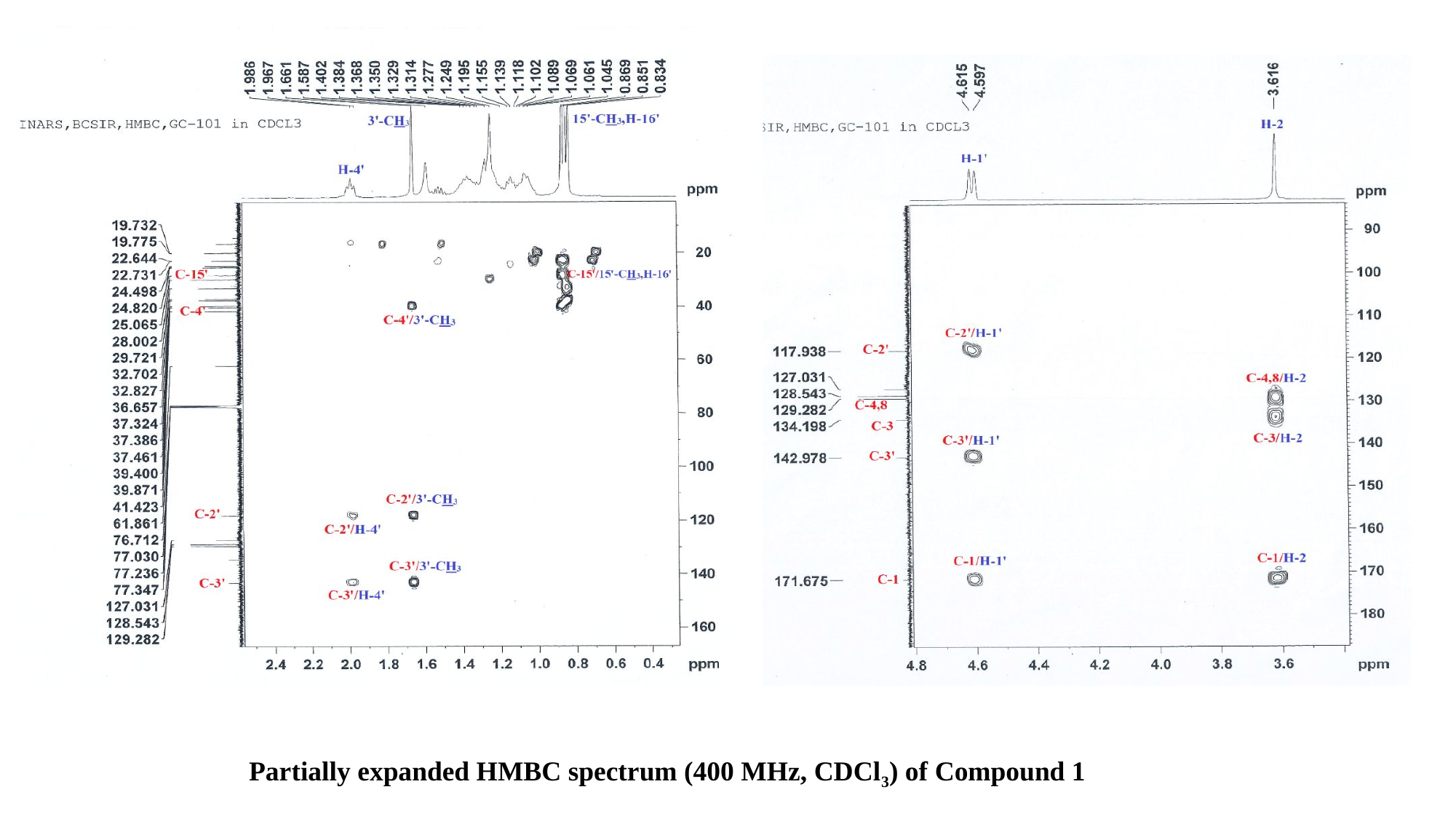

Partially expanded HMBC spectrum (400 MHz, CDCl3) of Compound 1

## Slide 6
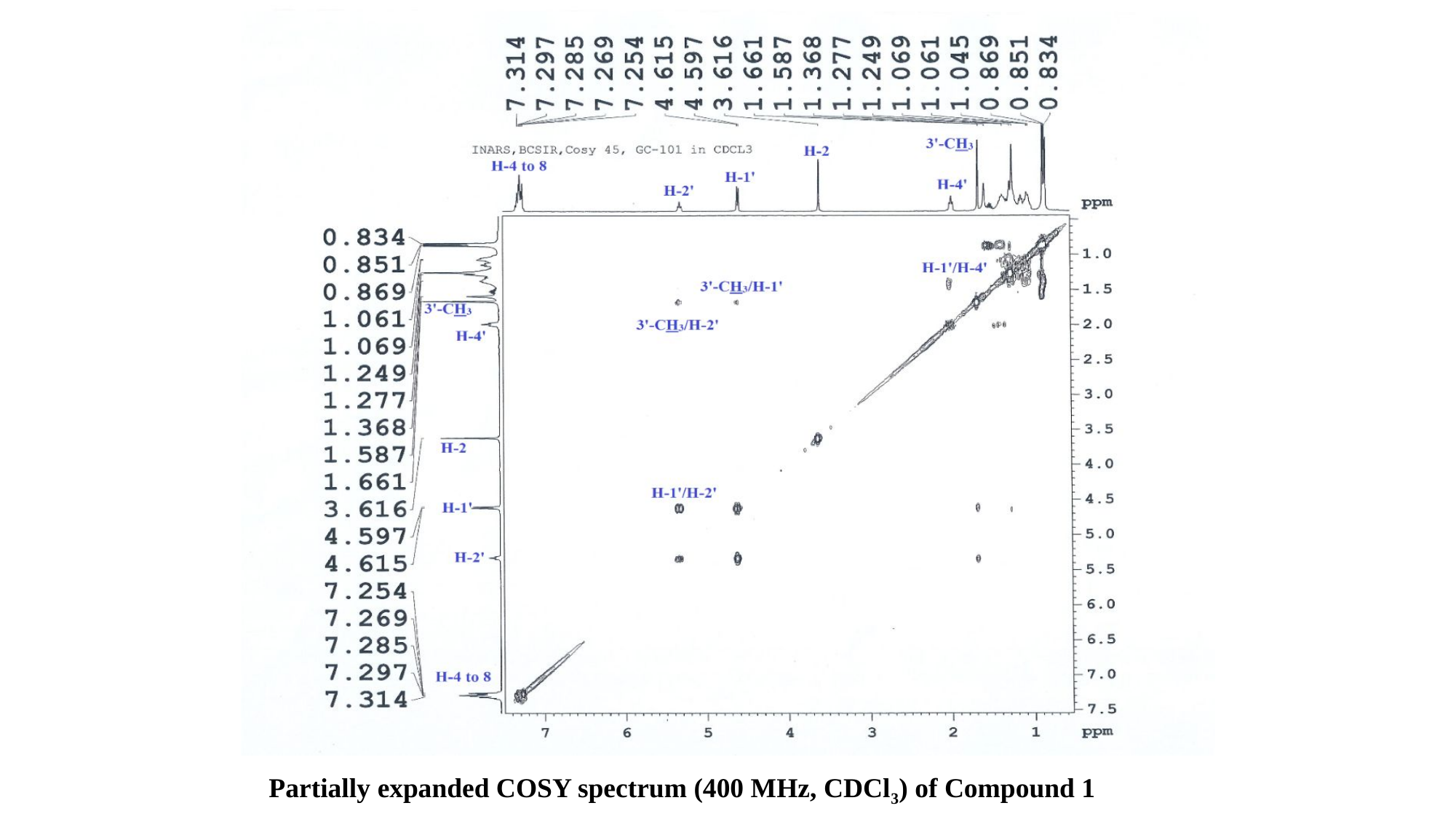

Partially expanded COSY spectrum (400 MHz, CDCl3) of Compound 1

## Slide 7
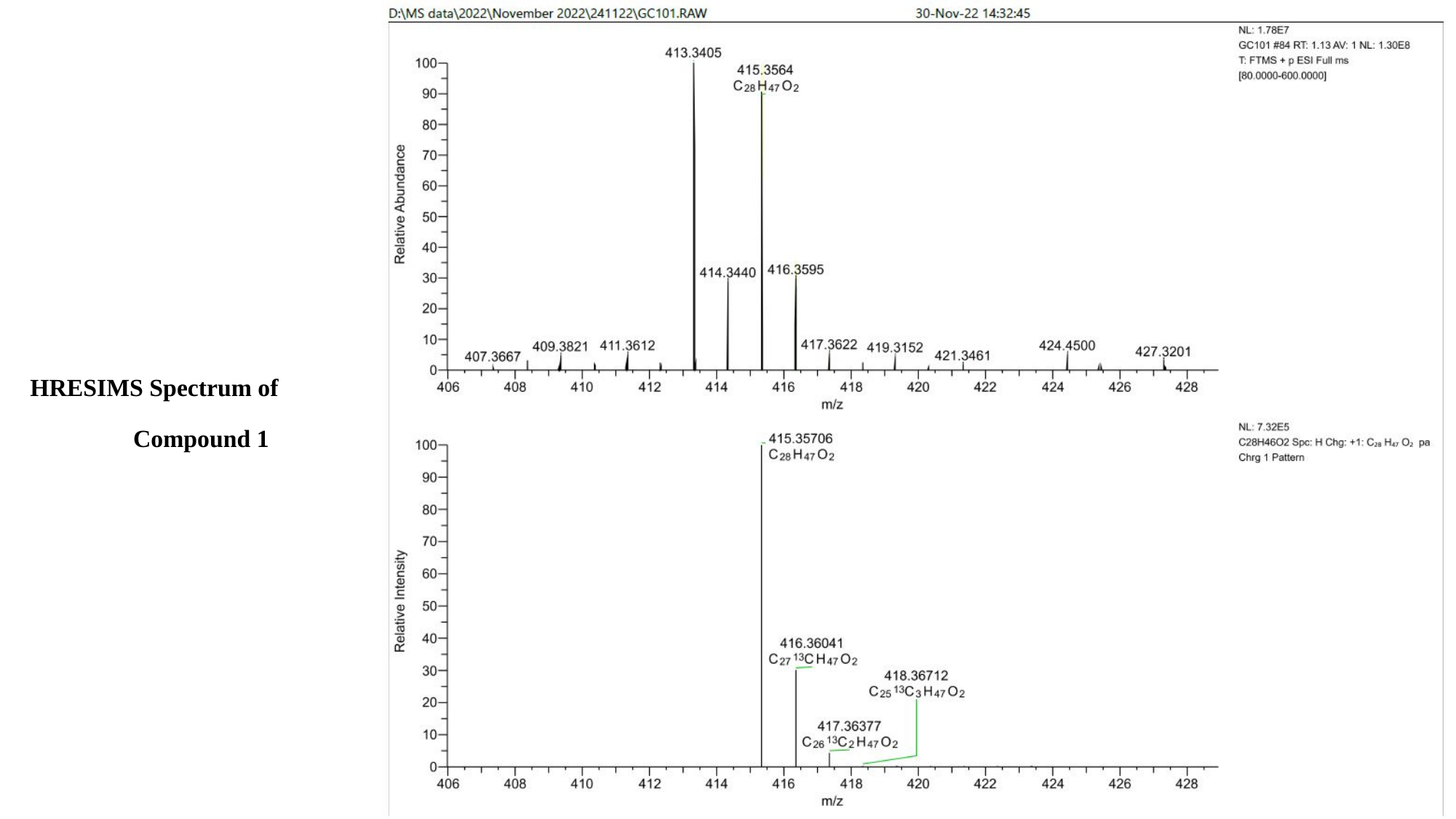

HRESIMS Spectrum of Compound 1

## Slide 8
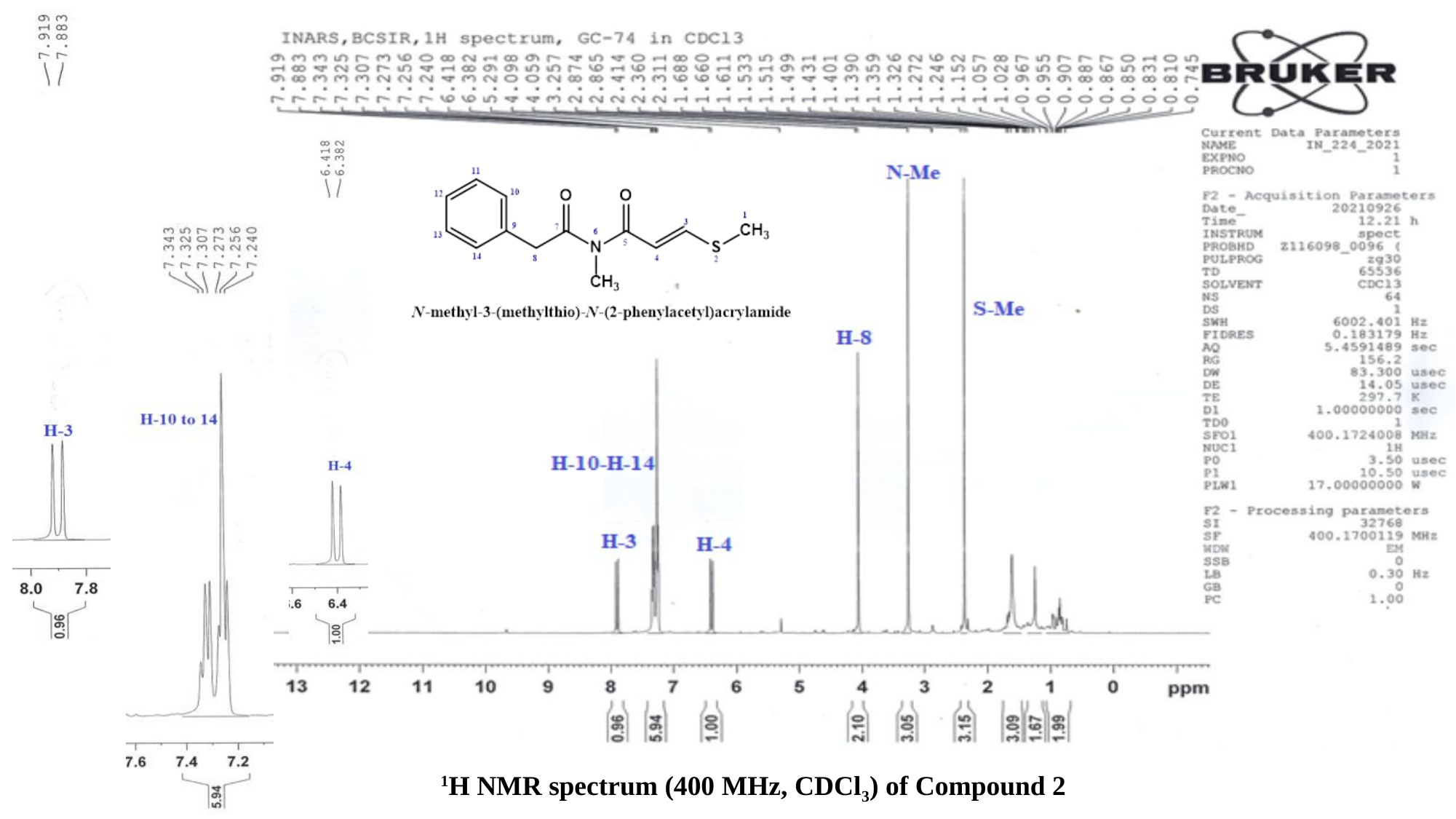

1H NMR spectrum (400 MHz, CDCl3) of Compound 2

## Slide 9
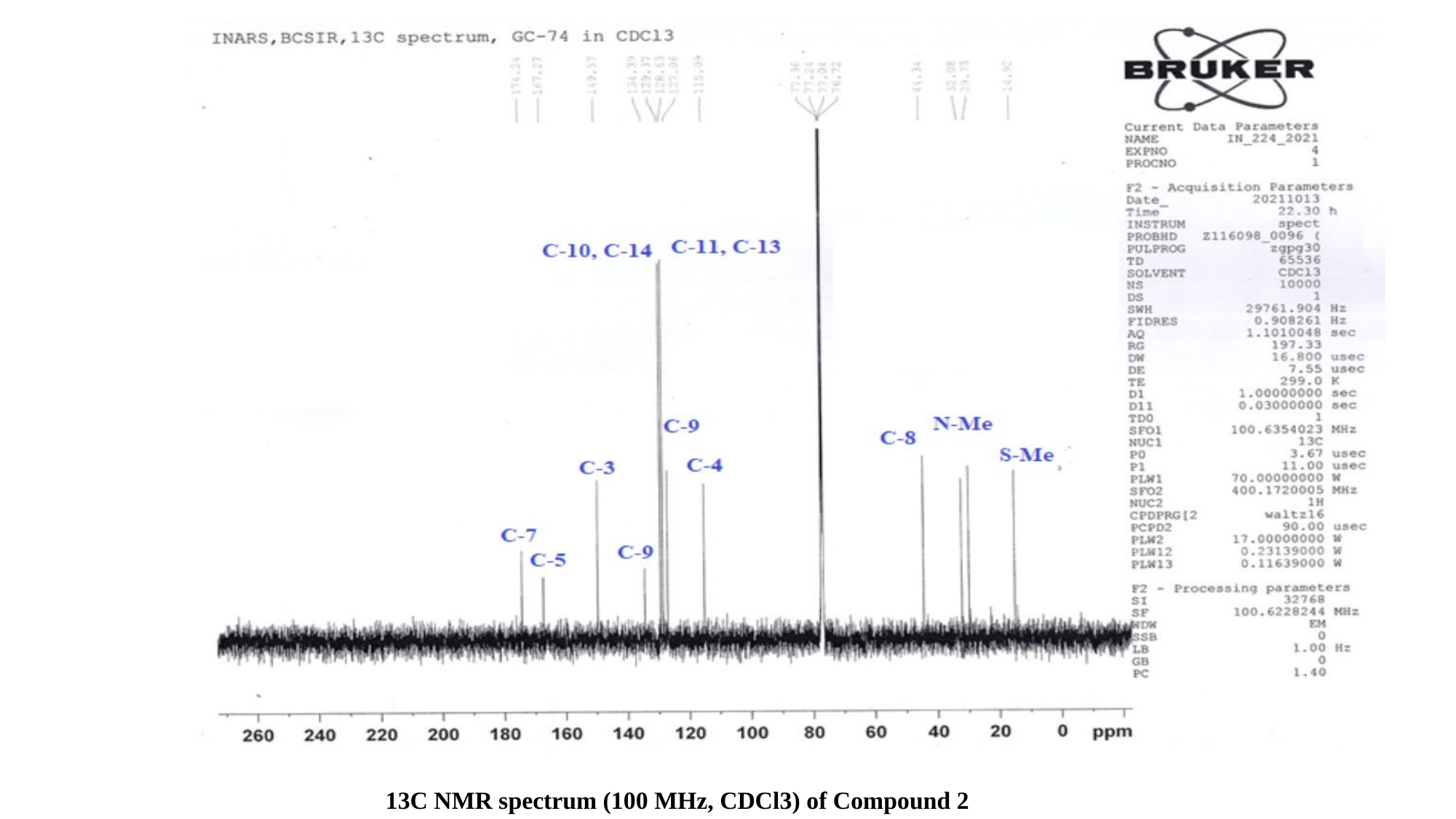

13C NMR spectrum (100 MHz, CDCl3) of Compound 2

## Slide 10
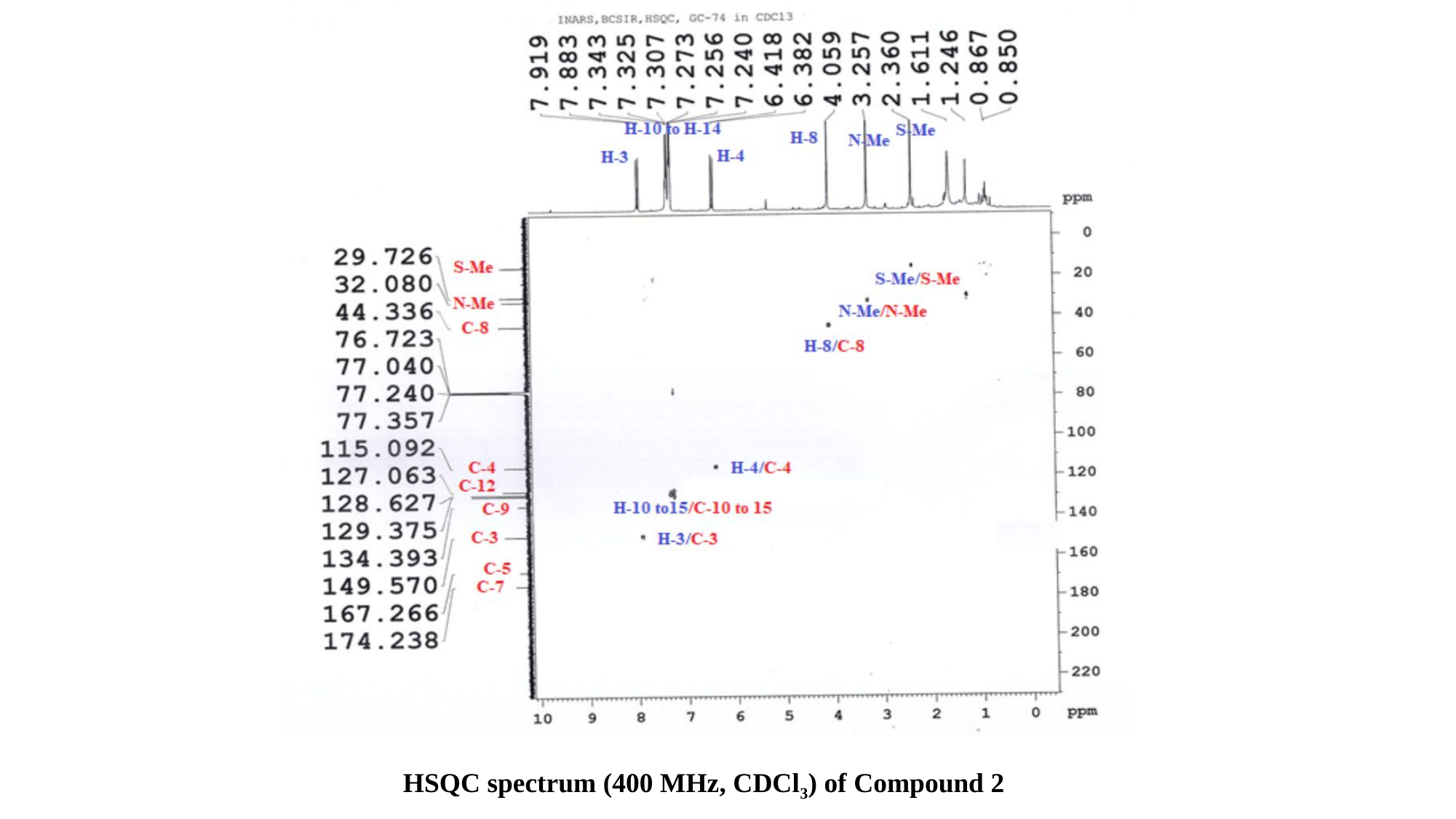

HSQC spectrum (400 MHz, CDCl3) of Compound 2

## Slide 11
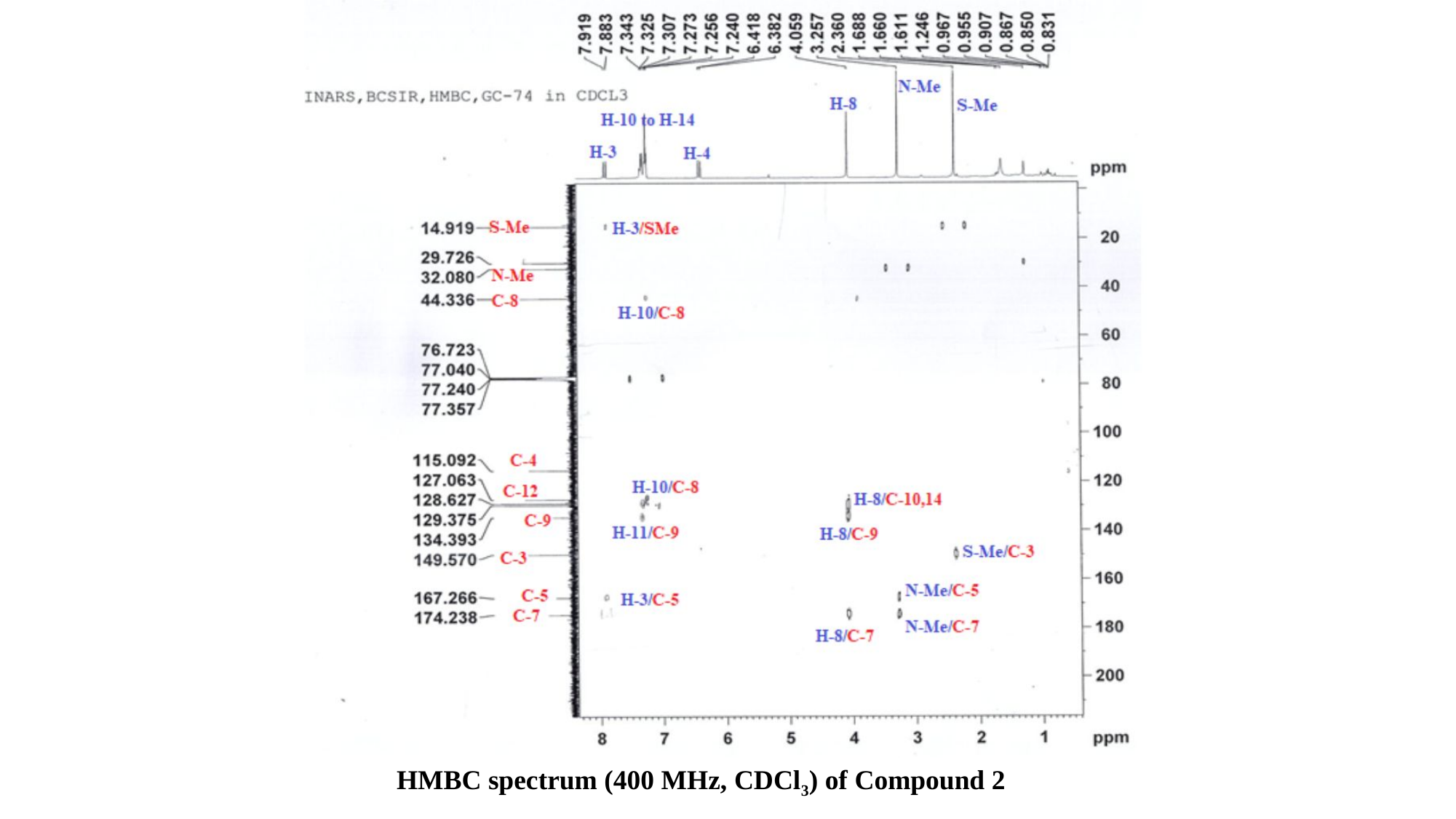

HMBC spectrum (400 MHz, CDCl3) of Compound 2

## Slide 12
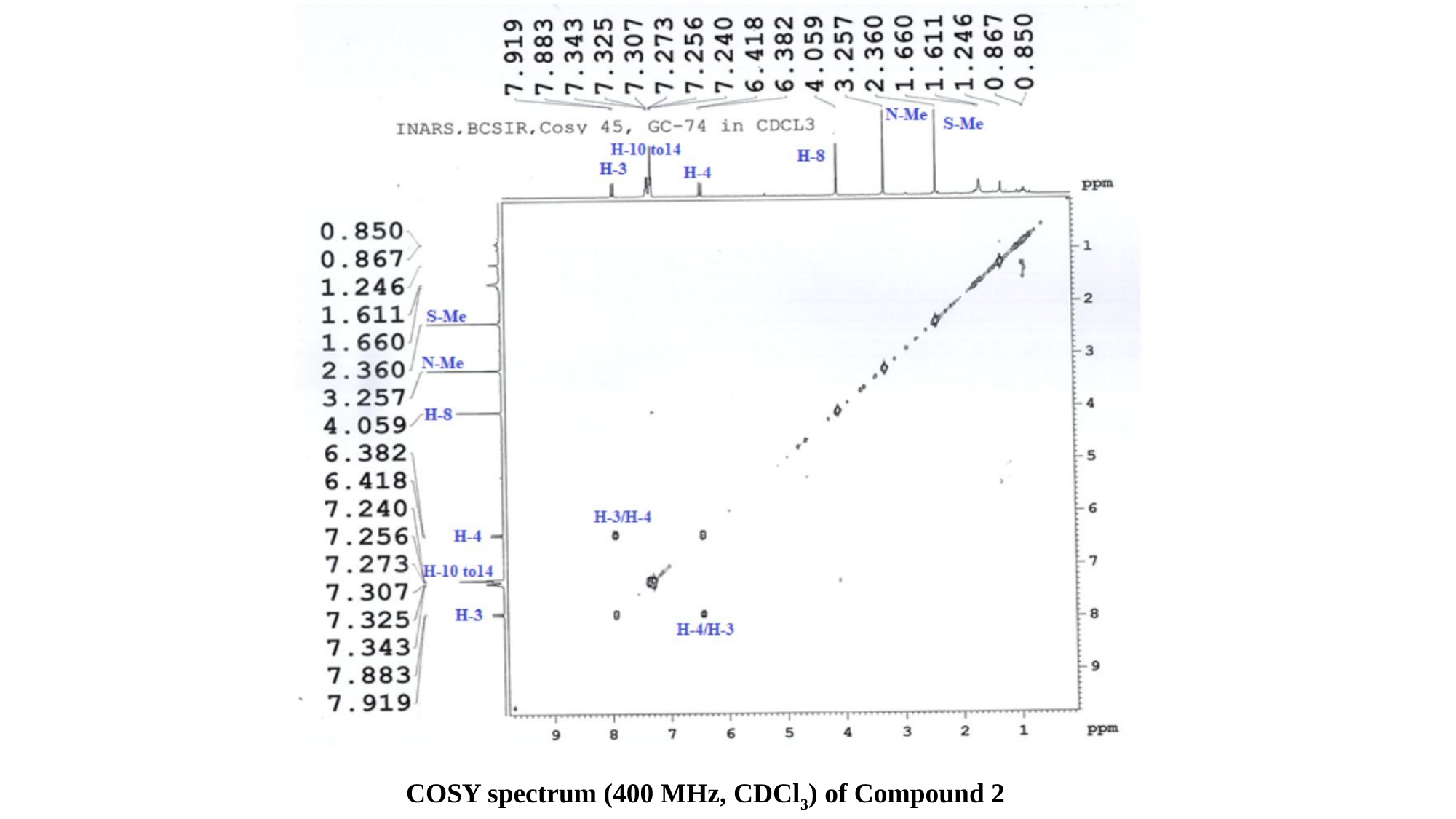

COSY spectrum (400 MHz, CDCl3) of Compound 2

## Slide 13
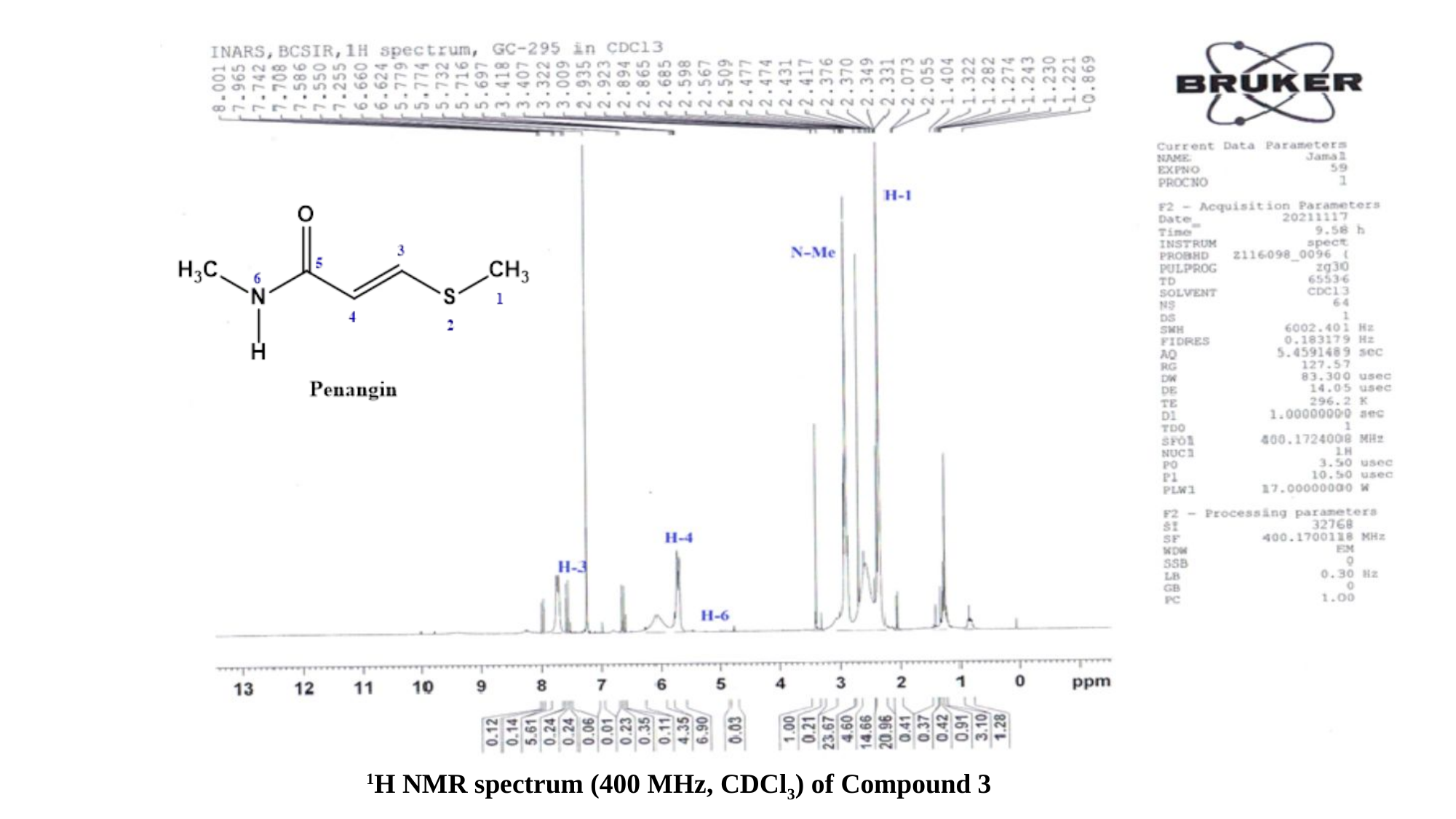

1H NMR spectrum (400 MHz, CDCl3) of Compound 3

## Slide 14
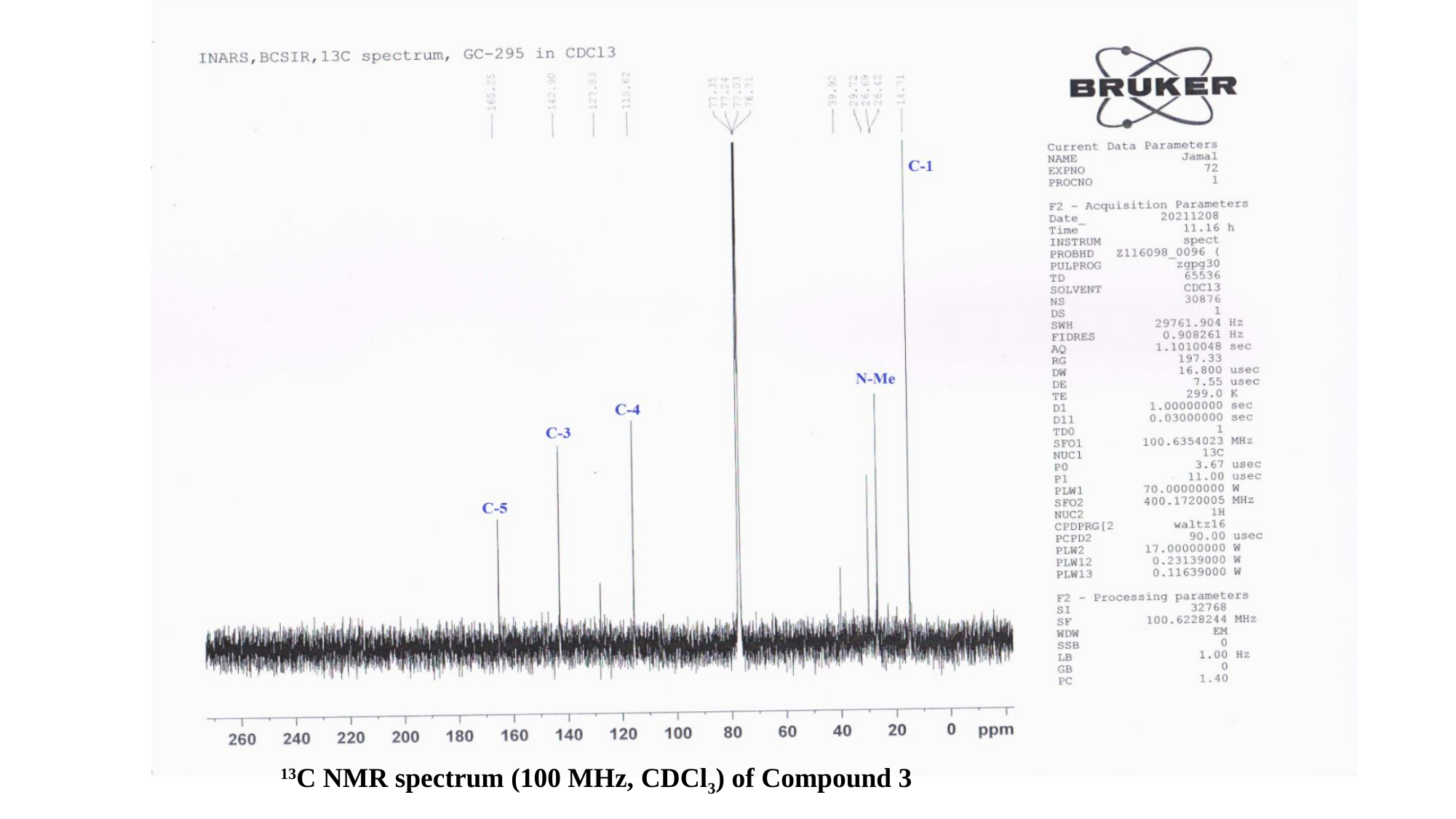

13C NMR spectrum (100 MHz, CDCl3) of Compound 3

## Slide 15
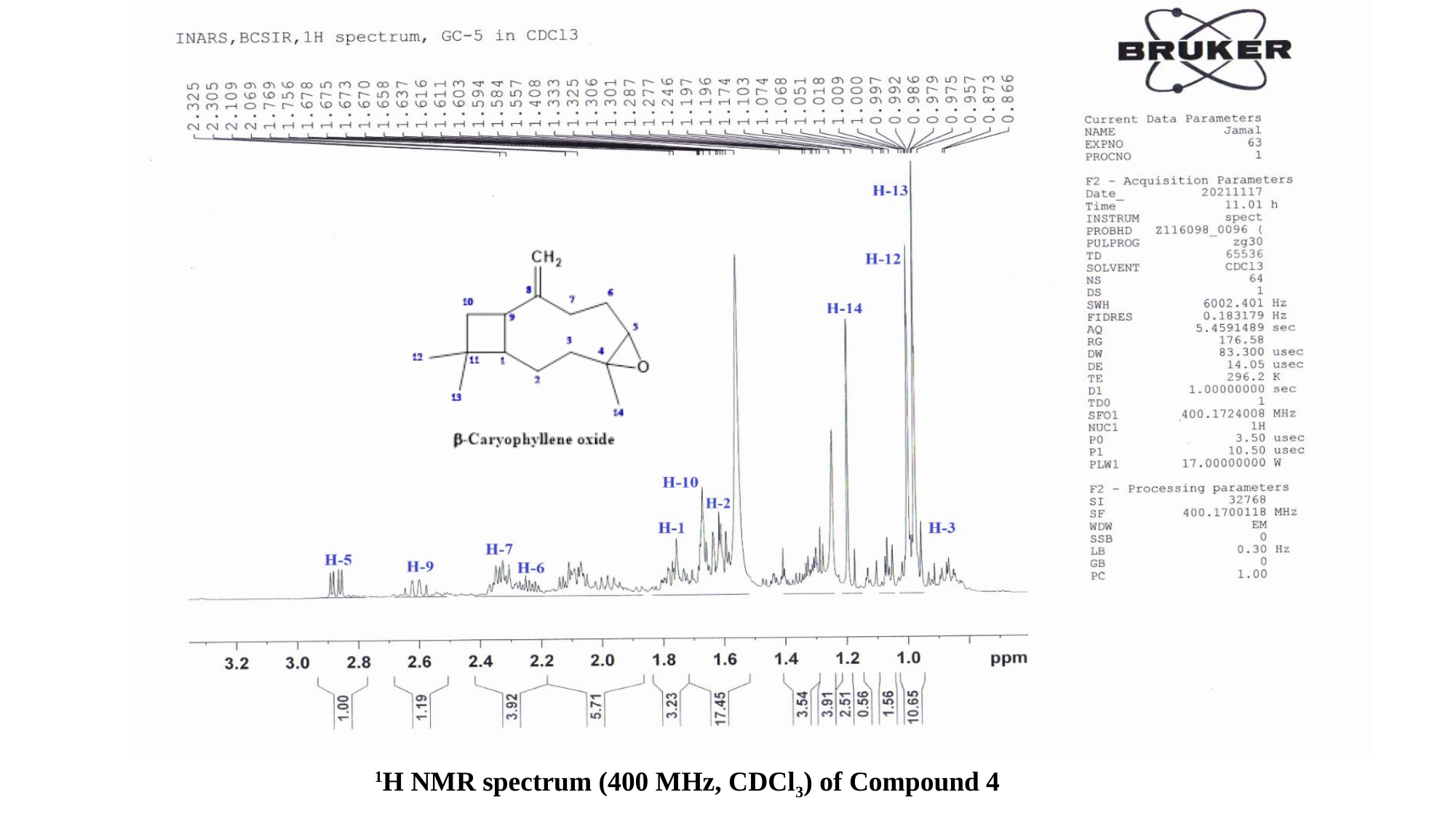

1H NMR spectrum (400 MHz, CDCl3) of Compound 4

## Slide 16
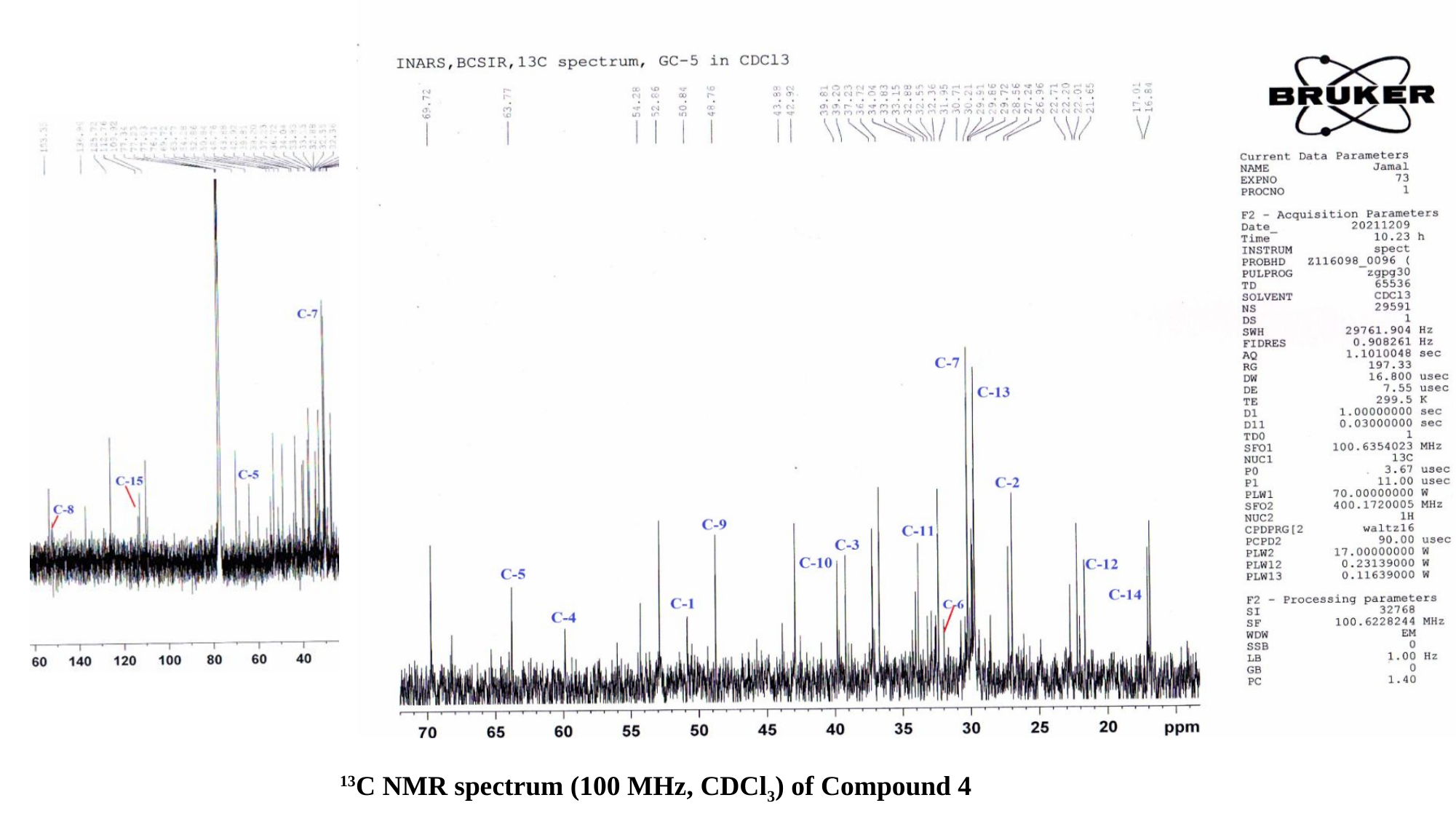

13C NMR spectrum (100 MHz, CDCl3) of Compound 4

## Slide 17
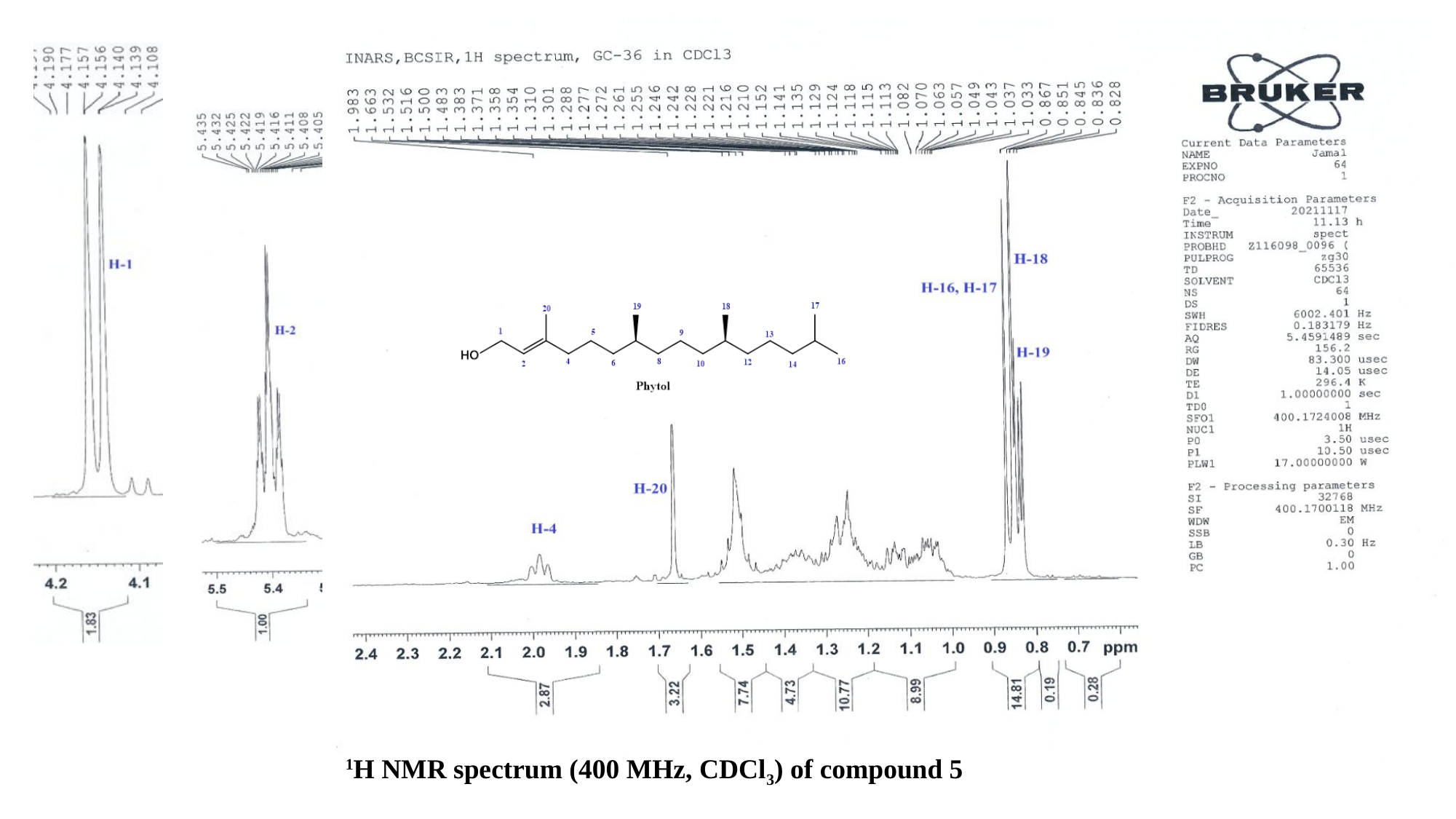

1H NMR spectrum (400 MHz, CDCl3) of compound 5

## Slide 18
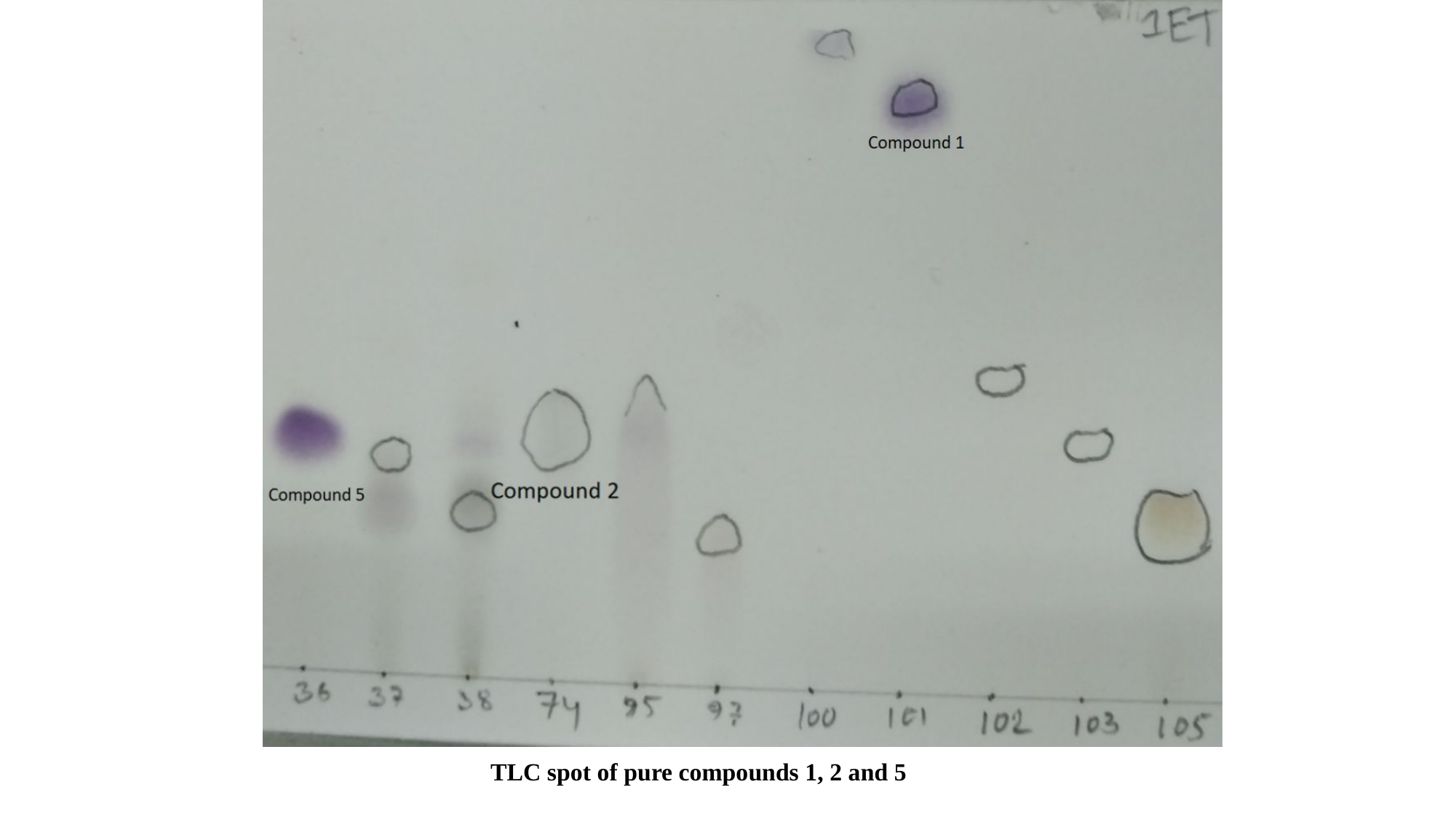

TLC spot of pure compounds 1, 2 and 5

## Slide 19
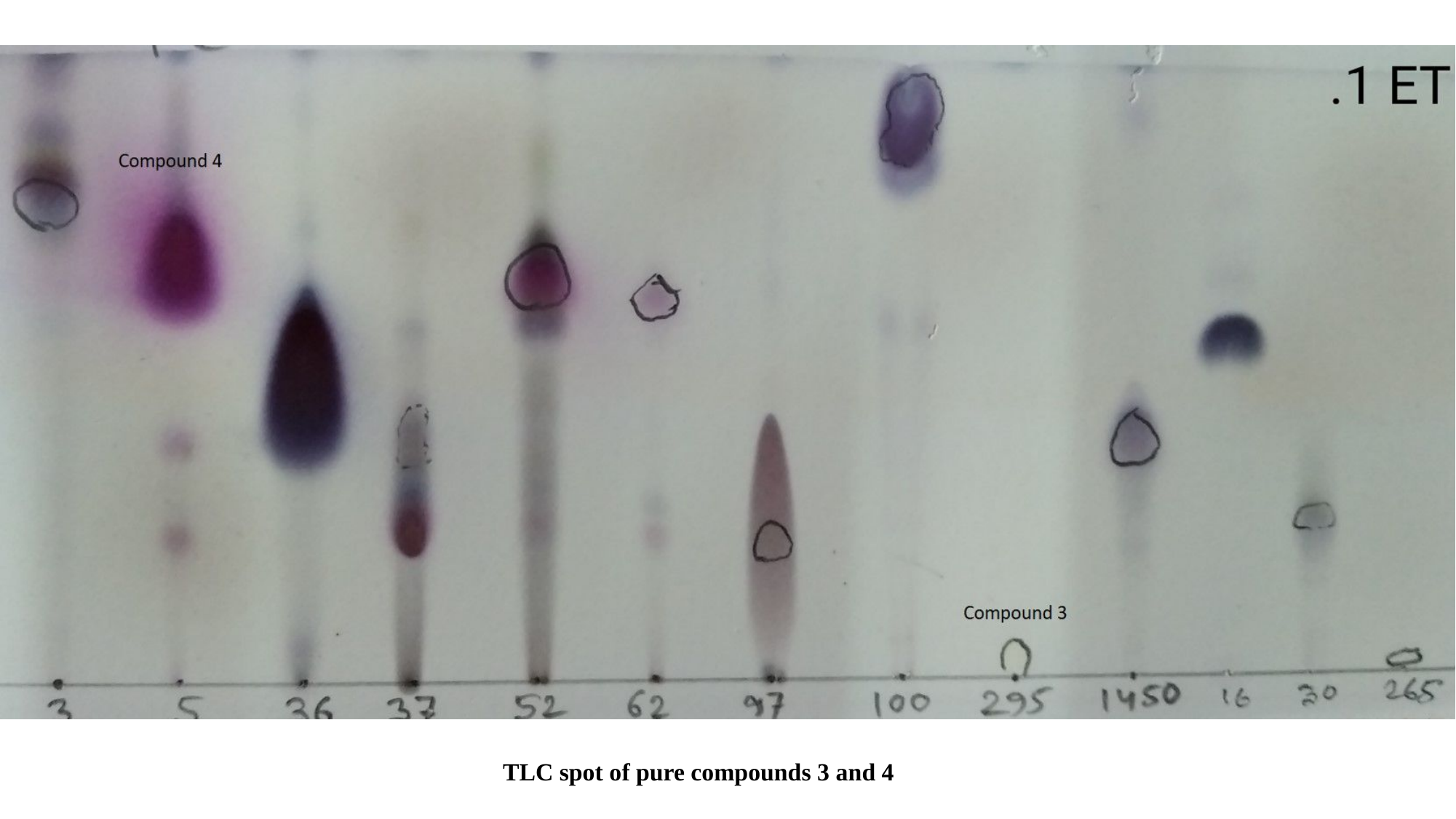

TLC spot of pure compounds 3 and 4
